# Supplementary material for: Nanoparticle delivery of a prodrug-activating bacterial enzyme leads to anti-tumor responses
Source: Nat Commun. 2025 Apr 12;16:3490. doi: 10.1038/s41467-025-58548-1 (PMC11993580; doi:10.1038/s41467-025-58548-1)
Supplement: Supplementary file 1 — Supplementary Information [file 41467_2025_58548_MOESM1_ESM.pdf]

Supplementary Information

Nanoparticle delivery of a prodrug-activating bacterial enzyme leads to anti-tumor responses

Sebastian G. Huayamares[1], Liming Lian[1], Regina Rab[2], Yuning Hou[3], Afsane Radmand[4,5], Hyejin Kim[1], Ryan Zenhausern[1], Bhagelu R. Achyut[2,3], Melissa Gilbert Ross[2,3], Melissa P. Lokugamage[1], David Loughrey[1], Hannah E. Peck[1], Elisa Schrader Echeverri[1], Alejandro J. Da Silva Sanchez[4,5], Aram Shajii[1], Andrea Li[1], Karen E. Tiegreen[1], Philip J. Santangelo[1], Eric J. Sorscher[2,3,*], James E. Dahlman[1,*]

[1]Wallace H. Coulter Department of Biomedical Engineering, Georgia Institute of Technology and Emory University School of Medicine, Atlanta, GA, 30332, USA

[2]Department of Pediatrics, Emory University, Atlanta, GA, USA

[3]Winship Cancer Institute, Emory University, Atlanta, GA, 30322, USA

[4]Petit Institute for Bioengineering and Biosciences, Georgia Institute of Technology, Atlanta, GA, 30332, USA

[5]Department of Chemical Engineering, Georgia Institute of Technology, Atlanta, GA, 30332, USA

*Correspondence: esorscher@emory.edu, james.dahlman@emory.edu

Inventory of Supporting Information:

Supplementary Table 1 | LNP screening compositions.

Supplementary Table 2 | Normalized delivery of the IT LNP screen *in vivo*.

Supplementary Fig. 1 | Human HNSCC flow cytometry gating.

Supplementary Fig. 2 | Enrichment of LNP characteristics influencing IT delivery.

Supplementary Fig. 3 | Characterization and efficacy of IT LNPs delivering NanoLuc- and aVHH-encoding mRNA.

Supplementary Fig. 4 | Transfection of NanoLuc and aVHH in PDX-carrying NSG mice.

Supplementary Fig. 5 | scRNA-seq analysis of PDX tumors treated with LNP^{IT} or PBS.

Supplementary Fig. 6 | Reactome pathway analysis for scRNA-seq data from LNP^{IT}.

Supplementary Fig. 7 | Off-target PNP activity by LNP^{IT}.

Supplementary Fig. 8 | Tumor volumes and body weights of PDX-carrying NSG mice treated with LNP^{IT} carrying PNP mRNA combined with fludarabine.

Supplementary Fig. 9 | Clinical scoring system.

Supplementary Fig. 10 | PDX models and prodrug kinetics.

Supplementary Fig. 11 | Synthesis of C12-200 stereopure isoforms.

Supplementary Fig. 12 | NMR data for C12-200 stereopure isoforms

Supplementary Fig. 13 | Flow cytometry panel.

a

LNP	Ionizable Lipid	Cholesterol	PEG	Helper	Ionizable Lipid	Cholesterol	PEG	Helper	Mass Ratio	Diameters (nm)	PDI
MC3	DLin-MC3-DMA	Cholesterol	C14PEG2K	DSPC	50	38.5	1.5	10	10	97.8	0.21
cKK-E12	cKK-E12	Cholesterol	C14PEG2K	DOPE	35	46.5	2.5	16	10	141.2	0.26
SM-102	SM-102	Cholesterol	DMG-PEG2K	DSPC	50	38.5	1.5	10	15	94.2	0.12
C12-200	C12-200	Cholesterol	C14PEG2K	DOPE	35	46.5	2.5	16	10	118	0.15

b

LNP	BC	Lipomer	Cholesterol	PEG	Helper	Cholesterol Charge	Lipomer	Cholesterol	PEG	Helper	Mass Ratio	Diameters (nm)	Included?
1	TGCCGCAG	KB-S9	Cholesterol	C18PEG2K	DOPE	Neutral	45	28	2	25	10	93.4	Y
2	GCCTCGGC	KB-S9	Cholesterol	C18PEG2K	DOPE	Neutral	35	46	2	17	10	62.5	Y
3	CTTGCAAG	KB-S9	Cholesterol	C18PEG2K	DOTAP	Neutral	45	28	2	25	10	160.4	Y
4	AATCATCC	KB-S9	Cholesterol	C18PEG2K	DOTAP	Neutral	35	46	2	17	10	154.3	Y
5	GTTCAGGC	KB-S9	DC-Cholesterol.HCL	C18PEG2K	DOPE	Positive	45	28	2	25	10	159.9	Y
6	AAGGATCG	KB-S9	DC-Cholesterol.HCL	C18PEG2K	DOPE	Positive	35	46	2	17	10	193	Y
7	CGTTGGCC	KB-S9	DC-Cholesterol.HCL	C18PEG2K	DOTAP	Positive	45	28	2	25	10	143.3	Y
8	GCCATGAC	KB-S9	DC-Cholesterol.HCL	C18PEG2K	DOTAP	Positive	35	46	2	17	10	174.8	Y
9	ACCATCGA	KB-S10	Cholesterol	C18PEG2K	DOPE	Neutral	45	28	2	25	10	153	Y
10	CGTAGAAT	KB-S10	Cholesterol	C18PEG2K	DOPE	Neutral	35	46	2	17	10	112.2	Y
11	TTCCATGC	KB-S10	Cholesterol	C18PEG2K	DOTAP	Neutral	45	28	2	25	10	121.3	Y
12	CTACCTAG	KB-S10	Cholesterol	C18PEG2K	DOTAP	Neutral	35	46	2	17	10	320.9	N
13	TATACTCC	KB-S10	DC-Cholesterol.HCL	C18PEG2K	DOPE	Positive	45	28	2	25	10	129.9	Y
14	ACTTGACC	KB-S10	DC-Cholesterol.HCL	C18PEG2K	DOPE	Positive	35	46	2	17	10	395.9	N
15	TATGGCAA	KB-S10	DC-Cholesterol.HCL	C18PEG2K	DOTAP	Positive	45	28	2	25	10	210.7	N
16	TCATAATT	KB-S10	DC-Cholesterol.HCL	C18PEG2K	DOTAP	Positive	35	46	2	17	10	214.1	N
17	ACCGGAAT	KB-S12	Cholesterol	C18PEG2K	DOPE	Neutral	45	28	2	25	10	131.8	Y
18	ACTATTAA	KB-S12	Cholesterol	C18PEG2K	DOPE	Neutral	35	46	2	17	10	116.8	Y
19	CCTGGTCA	KB-S12	Cholesterol	C18PEG2K	DOTAP	Neutral	45	28	2	25	10	210.1	N
20	CGTAACGA	KB-S12	Cholesterol	C18PEG2K	DOTAP	Neutral	35	46	2	17	10	132.3	Y
21	TGCGAGAA	KB-S12	DC-Cholesterol.HCL	C18PEG2K	DOPE	Positive	45	28	2	25	10	126	Y
22	CGCAATCC	KB-S12	DC-Cholesterol.HCL	C18PEG2K	DOPE	Positive	35	46	2	17	10	82.7	Y
23	GTTGACTA	KB-S12	DC-Cholesterol.HCL	C18PEG2K	DOTAP	Positive	45	28	2	25	10	131.2	Y
24	ACGGCCGC	KB-S12	DC-Cholesterol.HCL	C18PEG2K	DOTAP	Positive	35	46	2	17	10	97.2	Y
25	GAAGATTC	KB-S13	Cholesterol	C18PEG2K	DOPE	Neutral	45	28	2	25	10	211.1	N
26	GCGATCTA	KB-S13	Cholesterol	C18PEG2K	DOPE	Neutral	35	46	2	17	10	233.1	N
27	TGGTCGAC	KB-S13	Cholesterol	C18PEG2K	DOTAP	Neutral	45	28	2	25	10	428.2	N
28	GATCGGCG	KB-S13	Cholesterol	C18PEG2K	DOTAP	Neutral	35	46	2	17	10	150.7	Y
29	GGATGCGA	KB-S13	DC-Cholesterol.HCL	C18PEG2K	DOPE	Positive	45	28	2	25	10	226.2	N
30	ATAGCAGG	KB-S13	DC-Cholesterol.HCL	C18PEG2K	DOPE	Positive	35	46	2	17	10	161.2	Y
31	TGGTTCCA	KB-S13	DC-Cholesterol.HCL	C18PEG2K	DOTAP	Positive	45	28	2	25	10	158.6	N
32	TTACTATC	KB-S13	DC-Cholesterol.HCL	C18PEG2K	DOTAP	Positive	35	46	2	17	10	203.6	N
33	CTGGATGA	KB-R9	Cholesterol	C18PEG2K	DOPE	Neutral	45	28	2	25	10	243.7	N
34	GTAAGCTA	KB-R9	Cholesterol	C18PEG2K	DOPE	Neutral	35	46	2	17	10	96.5	Y
35	TCGAGGAC	KB-R9	Cholesterol	C18PEG2K	DOTAP	Neutral	45	28	2	25	10	85.5	Y
36	TCCGCATT	KB-R9	Cholesterol	C18PEG2K	DOTAP	Neutral	35	46	2	17	10	127.1	Y
37	GCGTTACT	KB-R9	DC-Cholesterol.HCL	C18PEG2K	DOPE	Positive	45	28	2	25	10	111.5	Y
38	AACCAATA	KB-R9	DC-Cholesterol.HCL	C18PEG2K	DOPE	Positive	35	46	2	17	10	216.7	N
39	CGGACCAG	KB-R9	DC-Cholesterol.HCL	C18PEG2K	DOTAP	Positive	45	28	2	25	10	234.7	N
40	TCGTCAGT	KB-R9	DC-Cholesterol.HCL	C18PEG2K	DOTAP	Positive	35	46	2	17	10	184.5	Y
41	GGTACCGT	KB-R10	Cholesterol	C18PEG2K	DOPE	Neutral	45	28	2	25	10	140.9	Y
42	CAGCCGCG	KB-R10	Cholesterol	C18PEG2K	DOPE	Neutral	35	46	2	17	10	80.5	Y
43	GATAAGAC	KB-R10	Cholesterol	C18PEG2K	DOTAP	Neutral	45	28	2	25	10	144.5	Y
44	CCGGCGCA	KB-R10	Cholesterol	C18PEG2K	DOTAP	Neutral	35	46	2	17	10	163.9	Y
45	TACGACTA	KB-R10	DC-Cholesterol.HCL	C18PEG2K	DOPE	Positive	45	28	2	25	10	240	N
46	TATTCGGC	KB-R10	DC-Cholesterol.HCL	C18PEG2K	DOPE	Positive	35	46	2	17	10	84.8	Y
47	GGTCTCGC	KB-R10	DC-Cholesterol.HCL	C18PEG2K	DOTAP	Positive	45	28	2	25	10	70.1	Y
48	GCTATAAT	KB-R10	DC-Cholesterol.HCL	C18PEG2K	DOTAP	Positive	35	46	2	17	10	244.8	N
49 / LNP^{IT}	CATGAGGT	KB-R12	Cholesterol	C18PEG2K	DOPE	Neutral	35	46	2	17	10	111.3	Y
50	GTACGGTT	KB-R12	Cholesterol	C18PEG2K	DOPE	Neutral	45	28	2	25	10	187.9	Y
51	AGCCGAAC	KB-R12	Cholesterol	C18PEG2K	DOTAP	Neutral	45	28	2	25	10	244.8	N
52	ATCTCAGC	KB-R12	Cholesterol	C18PEG2K	DOTAP	Neutral	35	46	2	17	10	171.3	Y
53	AGCCAGCT	KB-R12	DC-Cholesterol.HCL	C18PEG2K	DOPE	Positive	45	28	2	25	10	114.3	Y
54	GGCCAAGG	KB-R12	DC-Cholesterol.HCL	C18PEG2K	DOPE	Positive	35	46	2	17	10	100.4	Y
55	TCTGATTC	KB-R12	DC-Cholesterol.HCL	C18PEG2K	DOTAP	Positive	45	28	2	25	10	242.9	N
56	AGCTGCAA	KB-R12	DC-Cholesterol.HCL	C18PEG2K	DOTAP	Positive	35	46	2	17	10	74.7	Y
57	AGTATCCA	KB-R13	Cholesterol	C18PEG2K	DOPE	Neutral	45	28	2	25	10	224.4	N
58	CGCCAGTA	KB-R13	Cholesterol	C18PEG2K	DOPE	Neutral	35	46	2	17	10	791.7	N
59	CGACGGAG	KB-R13	Cholesterol	C18PEG2K	DOTAP	Neutral	45	28	2	25	10	110.9	Y
60	CTTAACCT	KB-R13	Cholesterol	C18PEG2K	DOTAP	Neutral	35	46	2	17	10	188.6	Y
61	CGGAACTT	KB-R13	DC-Cholesterol.HCL	C18PEG2K	DOPE	Positive	45	28	2	25	10	124.8	Y
62	GCGGCTGG	KB-R13	DC-Cholesterol.HCL	C18PEG2K	DOPE	Positive	35	46	2	17	10	198.6	Y
63	GTCTTGGA	KB-R13	DC-Cholesterol.HCL	C18PEG2K	DOTAP	Positive	45	28	2	25	10	268.8	N
64	TCGTCTTC	KB-R13	DC-Cholesterol.HCL	C18PEG2K	DOTAP	Positive	35	46	2	17	10	152.4	Y
Naked 1	TGCGGCGA												
Naked 2	CTCAAGAC												
Pool												131.65	

Supplementary Table 1 | LNP screening compositions. a, Composition (in molar %), diameter (nm), and PDI of the initial bioluminescence-based screen of LNPs with DLin-MC3-DMA, cKK-E12, SM-102, and C12-200 as ionizable lipids, intratumorally injected to FaDu HNSCC tumors in NU/J mice. **b,** Composition (in molar %) and mass ratios (w/w of lipid/nucleic acid) of the 64 LNPs

formulated, out of which 44 had diameters between 50 and 200 nm and were injected to FaDu HNSCC tumors in NU/J mice. The winner LNPs (28 and 49/LNP^{IT}) are highlighted in a red box. Reagent concentrations: stereopure ionizable lipids at 10 mg/mL, cholesterol or DC-cholesterol at 5 mg/mL, C18PEG2K at 5 mg/mL, helper lipid (DOPE or DOTAP) at 5 mg/mL, and nucleic acid (for screens, aVHH mRNA and DNA barcode in a 9/1 w/w ratio) at 1 mg/mL.

											Normalized Delivery by Tumors and Averaged				
LNP	BC	Lipomer	Cholesterol	PEG	Helper	Cholesterol Charge	Lipomer	Cholesterol	PEG	Helper	T1	T2	T3	T4	Average
49/LNP[IT]	CATGAGGT	KB-R12	Cholesterol	C18PEG2K	DOPE	Neutral	35	46	2	17	4.119901	4.724812	2.408941	3.480821	3.683619
20	CGTAACGA	KB-S12	Cholesterol	C18PEG2K	DOTAP	Neutral	35	46	2	17	3.437833	3.727778	4.369671	3.160525	3.673952
18	ACTATTAA	KB-S12	Cholesterol	C18PEG2K	DOPE	Neutral	35	46	2	17	4.469136	2.726946	2.941153	4.475983	3.653305
28	GATCGGCG	KB-S13	Cholesterol	C18PEG2K	DOTAP	Neutral	35	46	2	17	2.930187	3.53064	3.819649	3.396983	3.419365
50	GTACGGTT	KB-R12	Cholesterol	C18PEG2K	DOPE	Neutral	45	28	2	25	4.42192	2.528648	2.609399	4.066003	3.406493
60	CTTAACCT	KB-R13	Cholesterol	C18PEG2K	DOTAP	Neutral	35	46	2	17	2.687466	4.010703	3.582857	3.211674	3.373175
59	CGACGGAG	KB-R13	Cholesterol	C18PEG2K	DOTAP	Neutral	45	28	2	25	2.596934	3.459387	3.933517	2.870083	3.21498
10	CGTAGAAT	KB-S10	Cholesterol	C18PEG2K	DOPE	Neutral	35	46	2	17	4.044318	3.41721	1.850493	3.380923	3.173236
3	CTTGCAAG	KB-S9	Cholesterol	C18PEG2K	DOTAP	Neutral	45	28	2	25	2.213092	2.755091	3.62356	3.486362	3.019526
4	AATCATCC	KB-S9	Cholesterol	C18PEG2K	DOTAP	Neutral	35	46	2	17	2.824218	2.118477	3.292639	3.634829	2.967541
36	TCCGCATT	KB-R9	Cholesterol	C18PEG2K	DOTAP	Neutral	35	46	2	17	2.17412	3.051286	3.492058	2.393132	2.777649
57	AGTATCCA	KB-R13	Cholesterol	C18PEG2K	DOPE	Neutral	45	28	2	25	3.470348	2.249445	2.237391	3.06858	2.756441
34	GTAAGCTA	KB-R9	Cholesterol	C18PEG2K	DOPE	Neutral	35	46	2	17	2.390453	3.073523	2.324891	2.671117	2.614996
52	ATCTCAGC	KB-R12	Cholesterol	C18PEG2K	DOTAP	Neutral	35	46	2	17	2.448325	1.25446	3.42468	2.527668	2.413783
42	CAGCCGCG	KB-R10	Cholesterol	C18PEG2K	DOPE	Neutral	35	46	2	17	2.524954	2.397182	1.931594	2.674721	2.382113
2	GCCTCGGC	KB-S9	Cholesterol	C18PEG2K	DOPE	Neutral	35	46	2	17	4.014337	1.338807	0.913302	2.856316	2.28069
21	TGCGAGAA	KB-S12	DC-Cholesterol.HCL	C18PEG2K	DOPE	Positive	45	28	2	25	2.279116	2.614032	2.19857	1.96466	2.264094
9	ACCATCGA	KB-S10	Cholesterol	C18PEG2K	DOPE	Neutral	45	28	2	25	2.0071	3.044118	2.010657	1.988436	2.262578
44	CCGGCGCA	KB-R10	Cholesterol	C18PEG2K	DOTAP	Neutral	35	46	2	17	1.581697	2.694041	2.584908	2.075469	2.234029
13	TATACTCC	KB-S10	DC-Cholesterol.HCL	C18PEG2K	DOPE	Positive	45	28	2	25	2.358752	2.729337	1.485914	1.955012	2.132254
43	GATAAGAC	KB-R10	Cholesterol	C18PEG2K	DOTAP	Neutral	45	28	2	25	1.958153	2.247125	2.415481	1.898783	2.129885
41	GGTACCGT	KB-R10	Cholesterol	C18PEG2K	DOPE	Neutral	45	28	2	25	1.929404	2.537927	2.008273	2.019882	2.123872
7	CGTTGGCC	KB-S9	DC-Cholesterol.HCL	C18PEG2K	DOTAP	Positive	45	28	2	25	1.681387	2.079371	2.473153	1.99724	2.057788
61	CGGAACTT	KB-R13	DC-Cholesterol.HCL	C18PEG2K	DOPE	Positive	45	28	2	25	1.754938	2.600079	2.01244	1.743898	2.027839
56	AGCTGCAA	KB-R12	DC-Cholesterol.HCL	C18PEG2K	DOTAP	Positive	35	46	2	17	2.020669	1.900143	2.160319	1.946085	2.006804
6	AAGGATCG	KB-S9	DC-Cholesterol.HCL	C18PEG2K	DOPE	Positive	35	46	2	17	1.68051	2.642523	1.69599	1.858116	1.969285
40	TCGTCAGT	KB-R9	DC-Cholesterol.HCL	C18PEG2K	DOTAP	Positive	35	46	2	17	1.687913	2.186309	2.030508	1.743417	1.912037
37	GCGTTACT	KB-R9	DC-Cholesterol.HCL	C18PEG2K	DOPE	Positive	45	28	2	25	1.911033	1.396763	2.135299	1.996238	1.859833
11	TTCCATGC	KB-S10	Cholesterol	C18PEG2K	DOTAP	Neutral	45	28	2	25	1.763464	1.350541	2.63149	1.692417	1.859478
47	GGTCTCGC	KB-R10	DC-Cholesterol.HCL	C18PEG2K	DOTAP	Positive	45	28	2	25	1.726102	1.946893	1.930074	1.766242	1.842328
53	AGCCAGCT	KB-R12	DC-Cholesterol.HCL	C18PEG2K	DOPE	Positive	45	28	2	25	1.867428	2.100031	1.724823	1.560026	1.813077
64	TCGTCTTC	KB-R13	DC-Cholesterol.HCL	C18PEG2K	DOTAP	Positive	35	46	2	17	1.489691	1.986643	2.044802	1.551053	1.768047
1	TGCCGCAG	KB-S9	Cholesterol	C18PEG2K	DOPE	Neutral	45	28	2	25	2.538426	1.66938	0.737016	2.033627	1.744612
22	CGCAATCC	KB-S12	DC-Cholesterol.HCL	C18PEG2K	DOPE	Positive	35	46	2	17	1.61761	2.02818	1.76262	1.480198	1.722152
35	TCGAGGAC	KB-R9	Cholesterol	C18PEG2K	DOTAP	Neutral	45	28	2	25	1.898131	1.11055	1.775328	2.083302	1.716828
24	ACGGCCGC	KB-S12	DC-Cholesterol.HCL	C18PEG2K	DOTAP	Positive	35	46	2	17	1.661504	1.399591	2.088613	1.580984	1.682673
8	GCCATGAC	KB-S9	DC-Cholesterol.HCL	C18PEG2K	DOTAP	Positive	35	46	2	17	1.623494	1.154635	2.192996	1.725777	1.674226
62	GCGGCTGG	KB-R13	DC-Cholesterol.HCL	C18PEG2K	DOPE	Positive	35	46	2	17	1.427918	2.025368	1.649608	1.427149	1.632511
23	GTTGACTA	KB-S12	DC-Cholesterol.HCL	C18PEG2K	DOTAP	Positive	45	28	2	25	1.431746	1.536682	1.91295	1.425208	1.576647
46	TATTCGGC	KB-R10	DC-Cholesterol.HCL	C18PEG2K	DOPE	Positive	35	46	2	17	1.531177	1.491545	1.619489	1.494123	1.534083
5	GTTCAGGC	KB-S9	DC-Cholesterol.HCL	C18PEG2K	DOPE	Positive	45	28	2	25	1.725938	1.150869	1.524145	1.728101	1.532264
30	ATAGCAGG	KB-S13	DC-Cholesterol.HCL	C18PEG2K	DOPE	Positive	35	46	2	17	1.505575	1.309615	1.687807	1.492905	1.498975
54	GGCCAAGG	KB-R12	DC-Cholesterol.HCL	C18PEG2K	DOPE	Positive	35	46	2	17	1.339283	1.47774	1.524826	1.290517	1.408091
**Naked**	CTCAAGAC										1.234299	1.225573	1.226106	1.125415	1.202848

Supplementary Table 2 | Normalized delivery of the IT LNP screen *in vivo*. Composition and normalized delivery of the 44 LNPs and naked DNA barcode, injected intratumorally into each of the four FaDu-bearing NU/J mice as part of the screen. Sorted in descending order by averaged normalized delivery. The winner LNPs (28 and 49/LNP^{IT}) are highlighted in a red box.

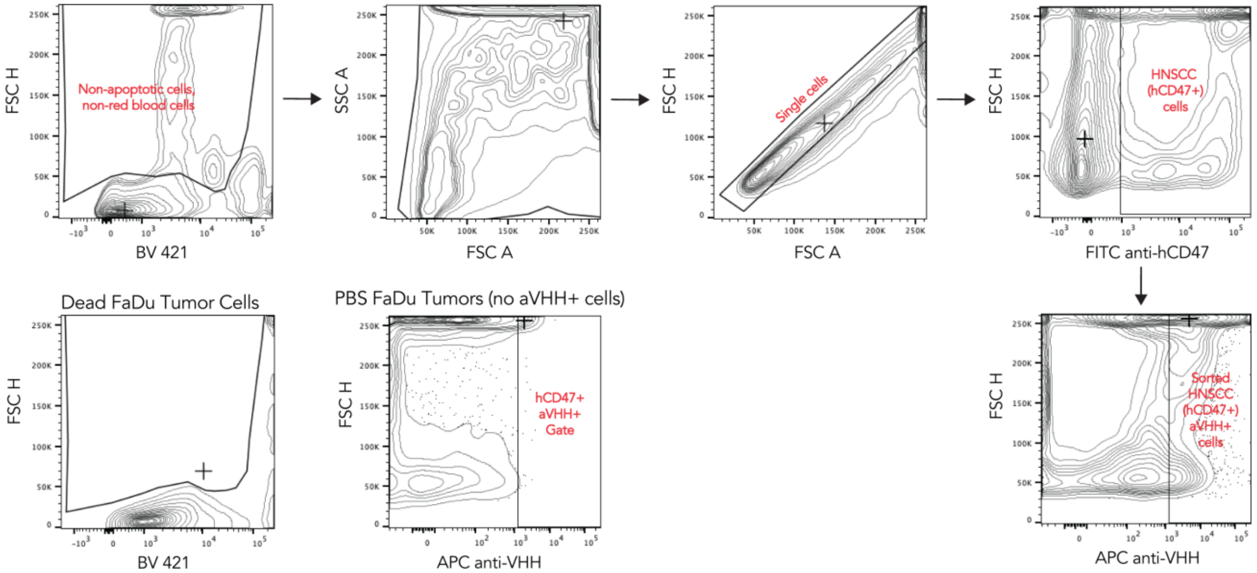

Supplementary Fig. 1 | Human HNSCC flow cytometry gating. Representative flow cytometry gates for isolating human HNSCC cells (FaDu or PDX) that were positively transfected with aVHH ($hCD47^+$ $aVHH^+$ cells), which were sorted for sequencing during the high-throughput SANDS screen.

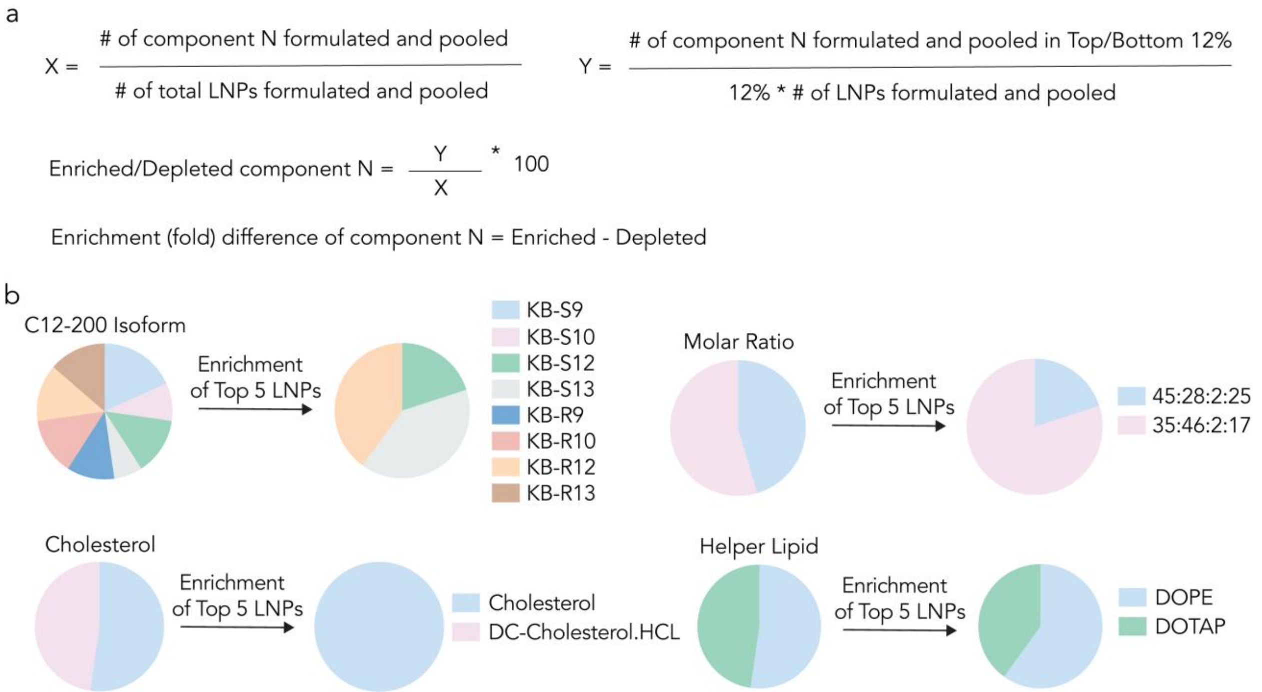

Supplementary Fig. 2 | Enrichment of LNP characteristics influencing IT delivery. a, Formula used to calculate the enrichment (fold) difference. **b,** Enrichment in the top 12% of LNPs (top 5 LNPs), subdivided by the ionizable lipid (C12-200 isoform), cholesterol type, molar ratio, and helper lipid used among the screened LNPs.

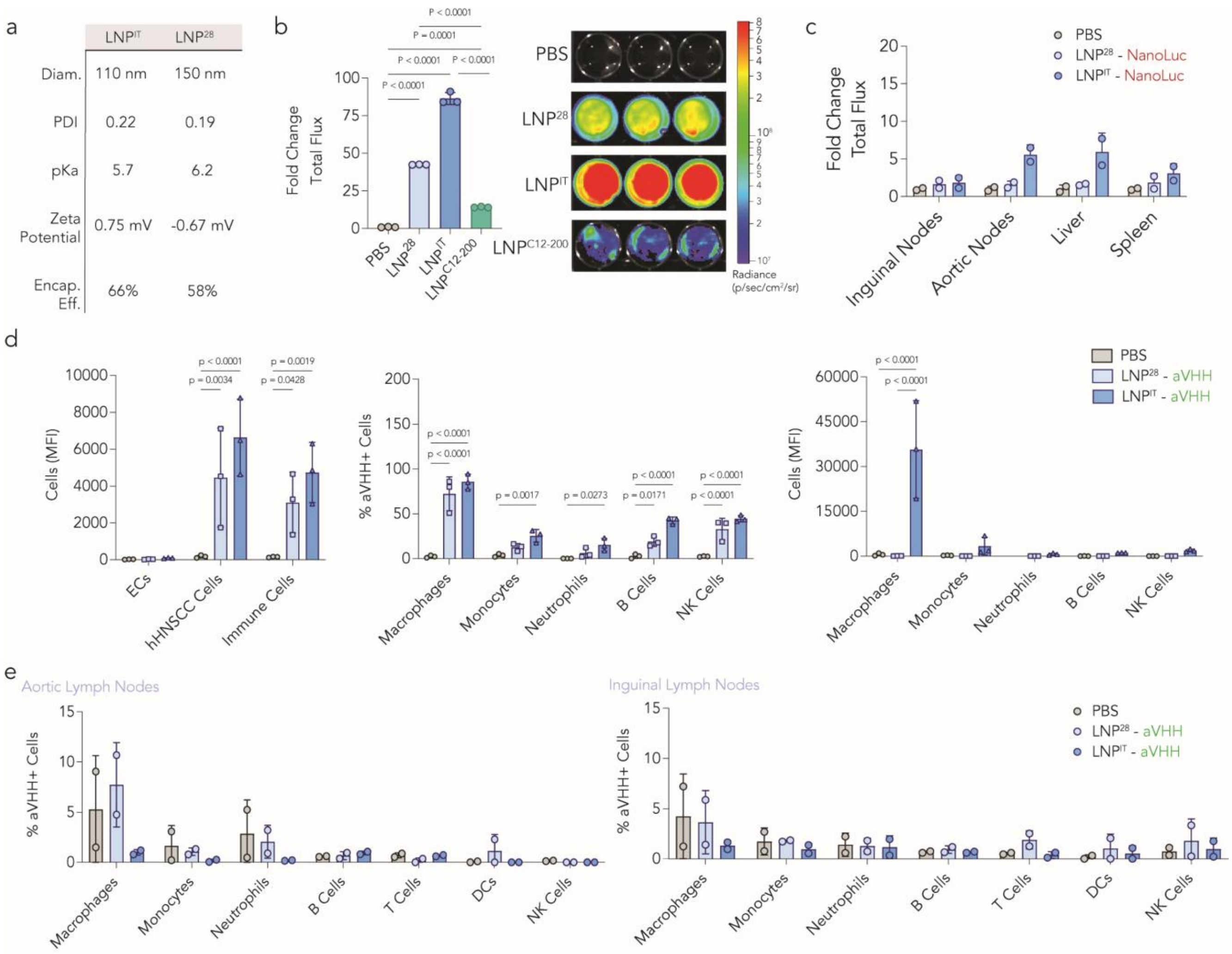

Supplementary Fig. 3 | Characterization and efficacy of IT LNPs delivering NanoLuc- and aVHH-encoding mRNA. a, Diameter (nm), polydispersity index, pKa, zeta potential (mV), and encapsulation efficiency (%) on LNP^{28} and LNP^{IT}. **b,** Both LNP^{28} and LNP^{IT} had higher transfection of FaDu cells than the LNP containing racemic C12-200 ($LNP^{C12\text{-}200}$) (n=3 experimental replicates consisting of wells seeded with the respective cell line, mean +/- SD). Data analyzed by ordinary one-way ANOVA. **c,** Bioluminescence measurements of off-target organs of NU/J mice injected with NanoLuc-carrying LNP^{28} and LNP^{IT} (n=2 experimental replicates, mean +/- SD). When injected with LNP^{28} and LNP^{IT} carrying aVHH mRNA, **d,** mean fluorescence intensity (MFI) was quantified in different cell types of the FaDu tumors (n=3 experimental replicates; mean +/- SD) analyzed by two-way ANOVA with Tukey post-hoc test. **e,** Off-target transfection was examined in the aortic and inguinal lymph nodes of NU/J mice (n=2 experimental replicates, mean +/- SD). ECs: endothelial cells, NK cells: natural killer cells, DCs: dendritic cells.

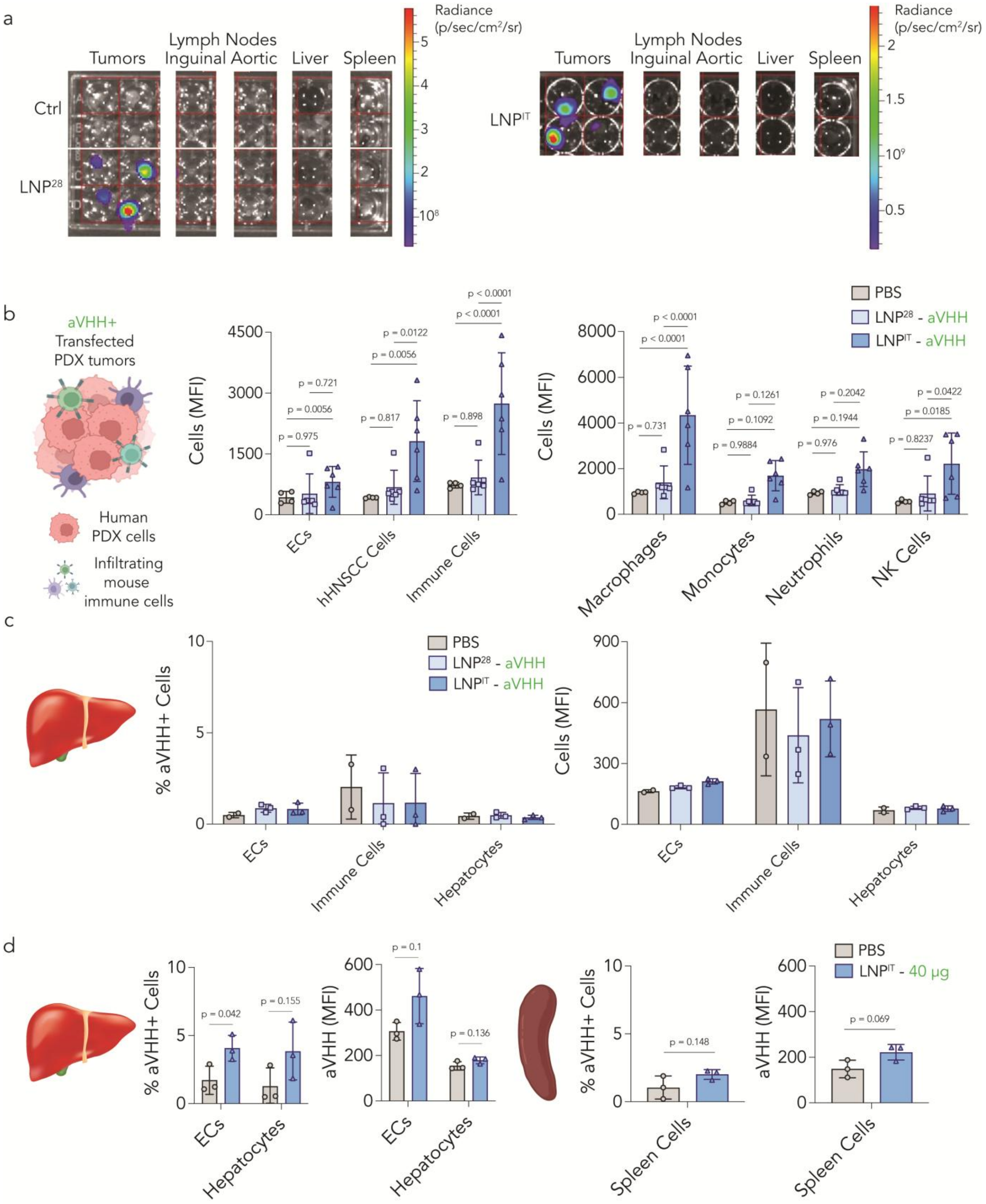

Supplementary Fig. 4 | Transfection of NanoLuc and aVHH in PDX-carrying NSG mice. a, Bioluminescence measurements of PDX tumors and off-target organs of NSG mice injected with

NanoLuc-carrying LNP[28] and LNP[IT]. When injected with LNP[28] and LNP[IT] carrying aVHH mRNA, **b,** MFI was quantified in the PDX tumors (n=4-6 experimental replicates; mean +/- SD) Data analyzed by two-way ANOVA with Tukey post-hoc test. **c,** Off-target transfection was examined in the livers of NSG mice (n=2-3 experimental replicates; mean +/- SD). **d,** Off-target transfection was profiled in the liver or spleen for LNP[IT] with LNP spin-concentrated to 40 µg/tumor (n=3 experimental replicates; mean +/- SD). Data analyzed by multiple unpaired t-test (Holm-Sidak with alpha =0.05) or two-tailed unpaired student's t-test. Sup. Fig. 4b,c,d were created with BioRender.com released under a Creative Commons Attribution-NonCommercial-NoDerivs 4.0 International license (https://creativecommons.org/licenses/by-nc-nd/4.0/deed.en).

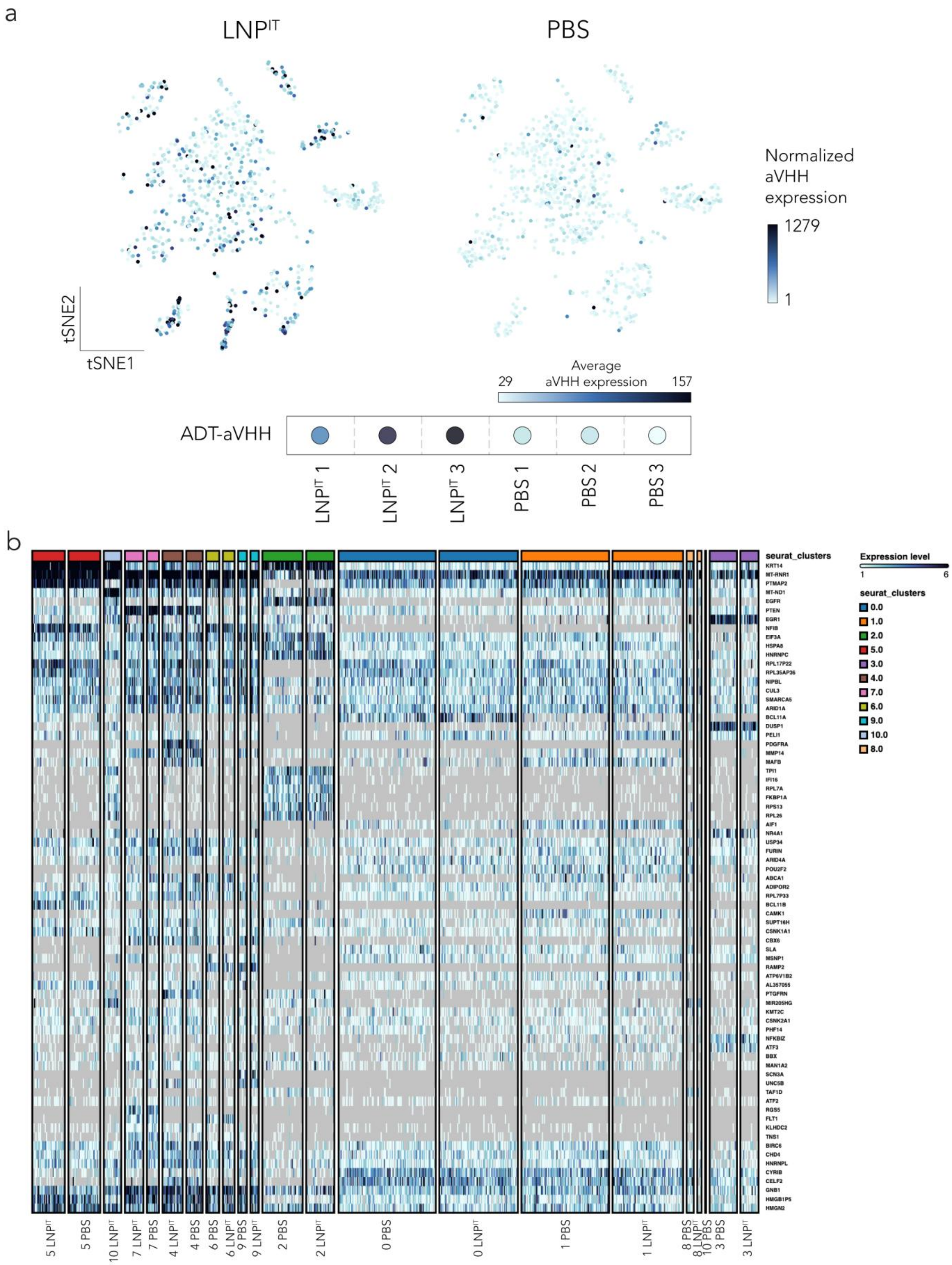
a
LNP^IT
PBS
Normalized aVHH expression
1279
1
tSNE2
tSNE1
Average aVHH expression
29
157
ADT-aVHH
LNP^IT 1
LNP^IT 2
LNP^IT 3
PBS 1
PBS 2
PBS 3
b
seurat_clusters
Expression level
1
6
seurat_clusters
0.0
1.0
2.0
5.0
3.0
4.0
7.0
6.0
9.0
10.0
8.0
KRT14
MT-RNR1
PTMAP2
MT-ND1
EGFR
PTEN
EGR1
NFIB
EIF3A
HSPA8
HNRNPC
RPL17P22
RPL35AP36
NIPBL
CUL3
SMARCA5
ARID1A
BCL11A
DUSP1
PELI1
PDGFRA
MMP14
MAFB
TPI1
IFI16
RPL7A
FKBP1A
RPS13
RPL26
AIF1
NR4A1
USP34
FURIN
ARID4A
POU2F2
ABCA1
ADIPOR2
RPL7P33
BCL11B
CAMK1
SUPT16H
CSNK1A1
CBX6
SLA
MSNP1
RAMP2
ATP6V1B2
AL357055
PTGFRN
MIR205HG
KMT2C
CSNK2A1
PHF14
NFKBIZ
ATF3
BBX
MAN1A2
SCN3A
UNC5B
TAF1D
ATF2
RGS5
FLT1
KLHDC2
TNS1
BIRC6
CHD4
HNRNPL
CYRIB
CELF2
GNB1
HMGB1P5
HMGN2
5 LNP^IT
5 PBS
10 LNP^IT
7 LNP^IT
7 PBS
4 LNP^IT
4 PBS
6 PBS
6 LNP^IT
9 PBS
9 LNP^IT
2 PBS
2 LNP^IT
0 PBS
0 LNP^IT
1 PBS
1 LNP^IT
8 PBS
8 LNP^IT
10 PBS
3 PBS
3 LNP^IT

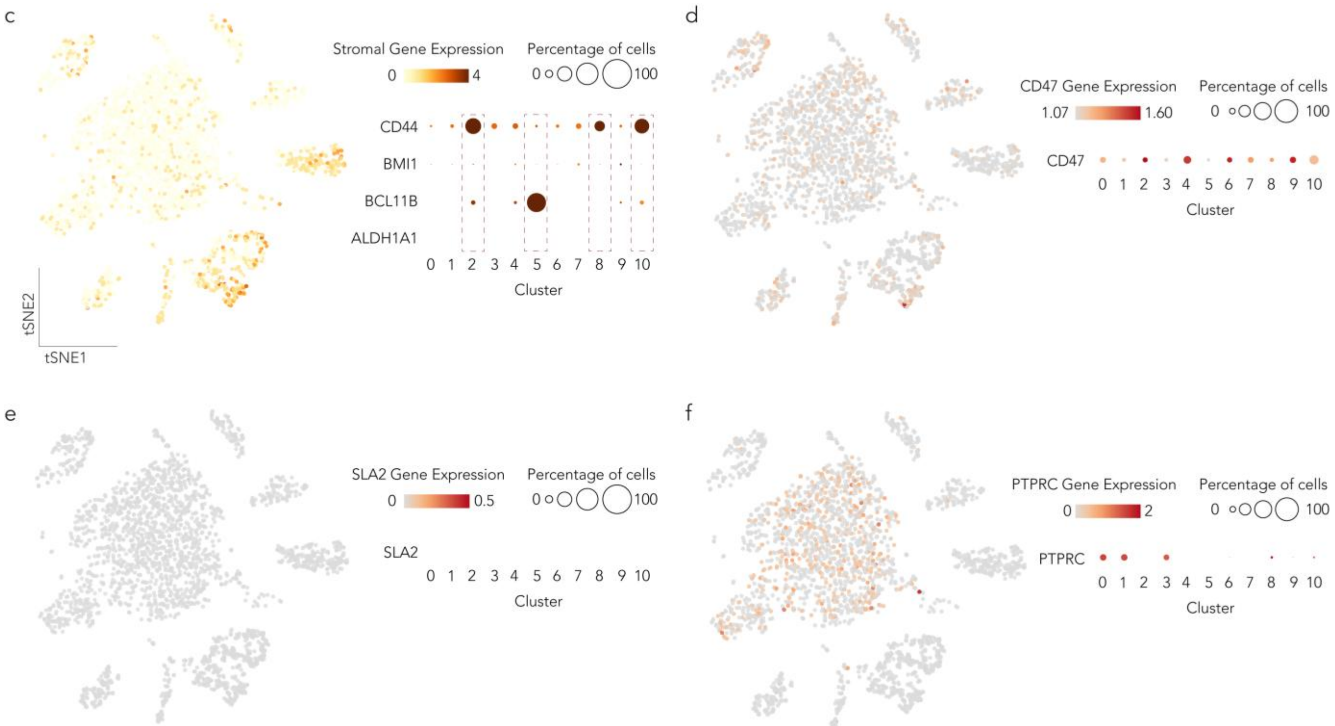

Supplementary Fig. 5 | scRNA-seq analysis of PDX tumors treated with LNP^{IT} or PBS. a, aVHH protein expression quantified via CITE-seq in PDX tumors treated with LNP^{IT} or PBS. **b,** Complete heatmap of genes expressed across all clusters in PDX tumors treated with LNP^{IT} or PBS. Expression levels of **c,** stromal genes, **d,** CD47, **e,** SLA2, and **f,** PTPRC (or CD45) across all clusters in the PDX tumors.

a

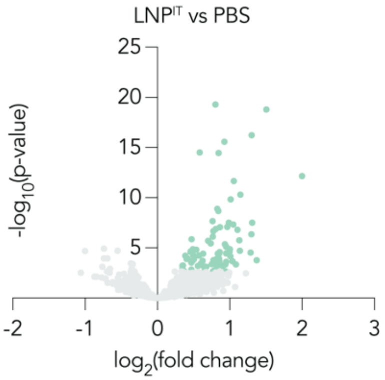

Top 100 Differential Genes, LNP^IT vs PBS:

Gene	Log2(FC)	-Log10(FDR)	FDR	P-value
CHMP4B	0.802283	19.291214	5.1143E-20	0.49777
ZNFX1	1.506194	18.7885208	1.62734E-19	0.039738
STAT1	1.30299	16.2364611	5.80148E-17	0.014489
TMSB10	0.927599	15.5786715	2.63833E-16	0.319897
H3-3B	0.585163	14.5198307	3.02113E-15	0.976399
PSME1	0.847556	14.4689927	3.39631E-15	0.832984
SOCS1	1.999457	12.1687543	6.78025E-13	0.993818
TDRD7	1.05864	11.6614253	2.18059E-12	0.120919
SLC25A22	1.146281	10.3064819	4.93762E-11	0.485294
FNDC3A	1.013696	9.83638981	1.45751E-10	0.038824
MAX	0.828013	8.89367346	1.2774E-09	0.976026
PSME2	0.84443	8.691503	2.03468E-09	0.327362
PELI1	0.764411	7.67312509	2.12263E-08	0.033371
ASCC3	0.98924	7.51564294	3.0504E-08	0.039213
ZUP1	1.311324	7.51550712	3.05136E-08	0.005666
AIDA	1.051466	7.32523322	4.72897E-08	0.804246
USP25	0.966008	7.0984679	7.97135E-08	8.84E-05
ETNK1	0.864615	7.06610009	8.58816E-08	0.003589
NAA20	0.806168	6.89501153	1.27347E-07	0.29387
CCND2	1.106129	6.80796075	1.55611E-07	0.731718
SNX2	0.773032	6.67598197	2.10872E-07	0.102873
TPPP3	1.299721	6.35381117	4.42781E-07	0.662956
VCPIP1	0.767314	6.13843974	7.27043E-07	0.732327
CALM1	0.473976	5.85981549	1.38097E-06	0.057524
MX1	1.133944	5.76062094	1.73532E-06	2.14E-05
TOMM70	0.700629	5.7158798	1.92362E-06	0.160365
MBD2	0.737325	5.45207175	3.53125E-06	0.137834
ILRUN	0.934704	5.36687469	4.2966E-06	0.09961
MT-ND4	0.94813	5.17979426	6.61007E-06	0.226589
MT-ATP6	0.953298	5.10850832	7.78918E-06	0.385687
SIRPA	-0.740589	4.93539742	1.16039E-05	0.314947
LAMP2	0.489892	4.87140975	1.34459E-05	0.007881
MT-CO3	1.00399	4.86290919	1.37117E-05	0.083641
AHNAK	0.53855	4.82416136	1.49913E-05	0.927073
RNF213	1.141034	4.72719719	1.87414E-05	0.604893
RNF130	-0.55048	4.71295432	1.93663E-05	0.794966
TGFBR1	-1.001571	4.70127128	1.98943E-05	0.003839
NSD3	0.460904	4.66987036	2.1386E-05	0.021413
MT-ND3	1.000271	4.63858034	2.29837E-05	0.388913
XRN2	0.459442	4.61941254	2.40208E-05	0.001504
GREM1	0.860267	4.57487567	2.66149E-05	0.063911
IFIH1	1.290157	4.52324364	2.99748E-05	0.026491
MT-CYB	0.952206	4.45156554	3.53537E-05	0.028143
MT-ND2	0.835559	4.4304105	3.71184E-05	0.059923
KLF6	0.632709	4.4304105	3.71184E-05	0.304795
MT-ND1	0.852392	4.42638679	3.74639E-05	0.001929
RAB5C	0.494528	4.28137251	5.23152E-05	0.01898
S100A10	0.5756	4.23978444	5.75726E-05	0.330891
TMBIM6	0.384327	4.22243813	5.99186E-05	0.674859
MT-CO2	1.010467	4.1689051	6.7779E-05	4.22E-05

Gene	Log2(FC)	-Log10(FDR)	FDR	P-value
PTMS	0.416178	3.955736557	0.000111	0.337746
SLA	-0.693617	3.955736557	0.000111	0.898769
TRPS1	-0.739744	3.955736557	0.000111	0.781821
C6ORF62	0.403804	3.91413806	0.000122	0.218533
TMSB10P1	0.853336	3.91413806	0.000122	0.172079
AKT3	0.815642	3.890737097	0.000129	0.466462
HSP90AB3I	0.50457	3.879216104	0.000132	0.020257
LY6E	1.372825	3.779765161	0.000166	0.008533
DCP2	0.772958	3.773641089	0.000168	0.0019
TMEM184E	0.625972	3.73464878	0.000184	0.342161
S100A6	0.486774	3.714410382	0.000193	9.34E-05
STK40	1.04025	3.673176541	0.000212	0.897115
UBR4	0.856249	3.64328596	0.000227	0.639064
MIER3	0.950823	3.60964651	0.000246	0.037613
DYNLL1	0.470672	3.527507311	0.000297	0.129536
GDI2	-0.367051	3.474187692	0.000336	0.777578
FRMD4A	0.601216	3.426208999	0.000375	0.446144
PSME2P1	0.833697	3.426208999	0.000375	0.004907
PABPC1	-0.340016	3.379311225	0.000418	0.051196
SOS1	0.750502	3.379311225	0.000418	0.069248
FAM184B	1.087239	3.368622416	0.000428	0.859782
NAMPTP1	0.939865	3.346952786	0.00045	0.231168
GNB1	0.352628	3.254401655	0.000557	0.932255
UBB	0.344362	3.217619467	0.000606	0.000265
AFTPH	0.559072	3.202469967	0.000627	0.168489
AC022306	0.82547	3.202469967	0.000627	0.678397
N4BP1	0.634818	3.202469967	0.000627	0.017255
NAMPT	0.90532	3.197101508	0.000635	0.399811
TENT5A	0.676016	3.118490569	0.000761	0.098853
RMND5A	-0.760864	3.112195569	0.000772	0.003099
MT-ND5	0.662869	3.104147204	0.000787	0.968359
UBE2L3	0.344036	2.9436448	0.001139	0.37715
AC246787	-0.355807	2.9436448	0.001139	0.054399
POU2F2	-0.653888	2.906317279	0.001241	0.281564
ELF1	0.469078	2.837982448	0.001452	0.071895
MT-ND4L	0.787862	2.749833091	0.001779	0.546259
EHBP1-AS1	0.368847	2.690709359	0.002038	0.263817
SKAP2	-0.694725	2.662099814	0.002177	2.17E-05
VPS54	0.648296	2.662099814	0.002177	0.005171
SLCO3A1	0.766473	2.662099814	0.002177	0.153284
MTND1P23	0.9432	2.662099814	0.002177	0.810513
AC010343	-0.368631	2.662099814	0.002177	0.497574
ACTG1	0.239854	2.662099814	0.002177	2.3E-07
MT-RNR1	0.333008	2.662099814	0.002177	0.380614
RPL17P36	-0.315958	2.662099814	0.002177	3.42E-10
GRN	0.860926	2.658702825	0.002194	0.993697
G3BP2	0.352467	2.626449057	0.002363	0.298918
ZNF800	0.56547	2.603626934	0.002491	0.612121
HMGN3	0.672507	2.597229074	0.002528	0.769552
IGF2R	0.994482	2.552540433	0.002802	0.629876

Full Reactome Pathway Analysis, LNP^IT vs PBS ($p < 0.01$):

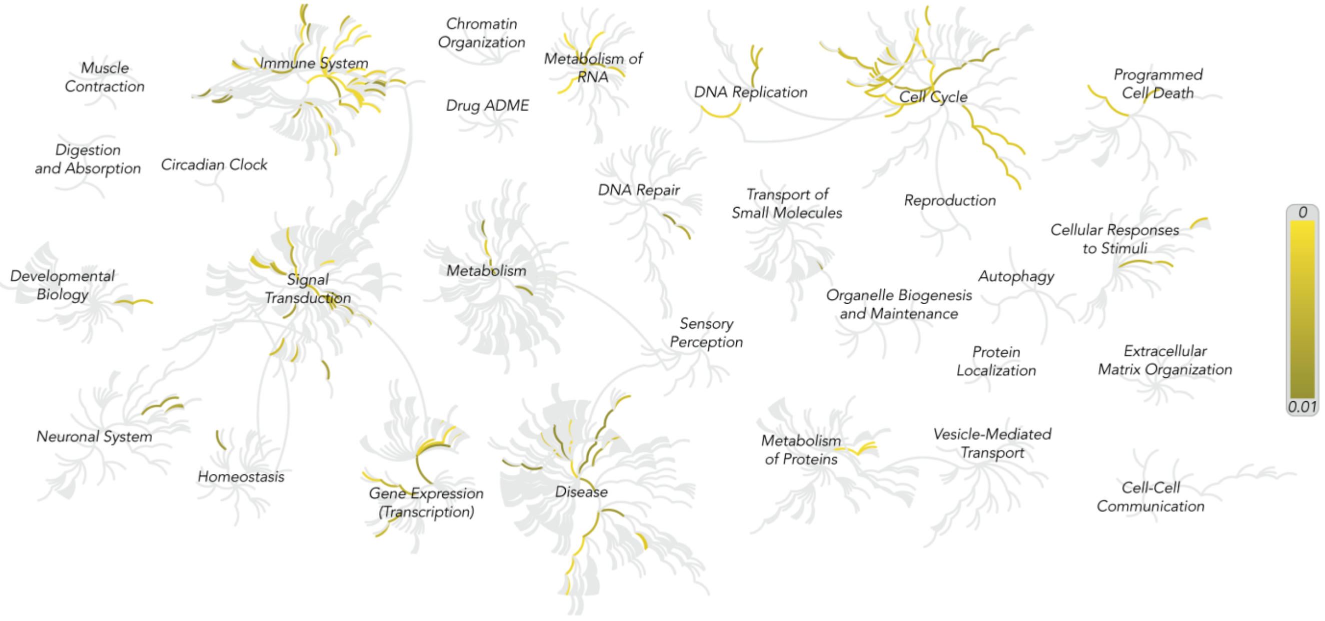

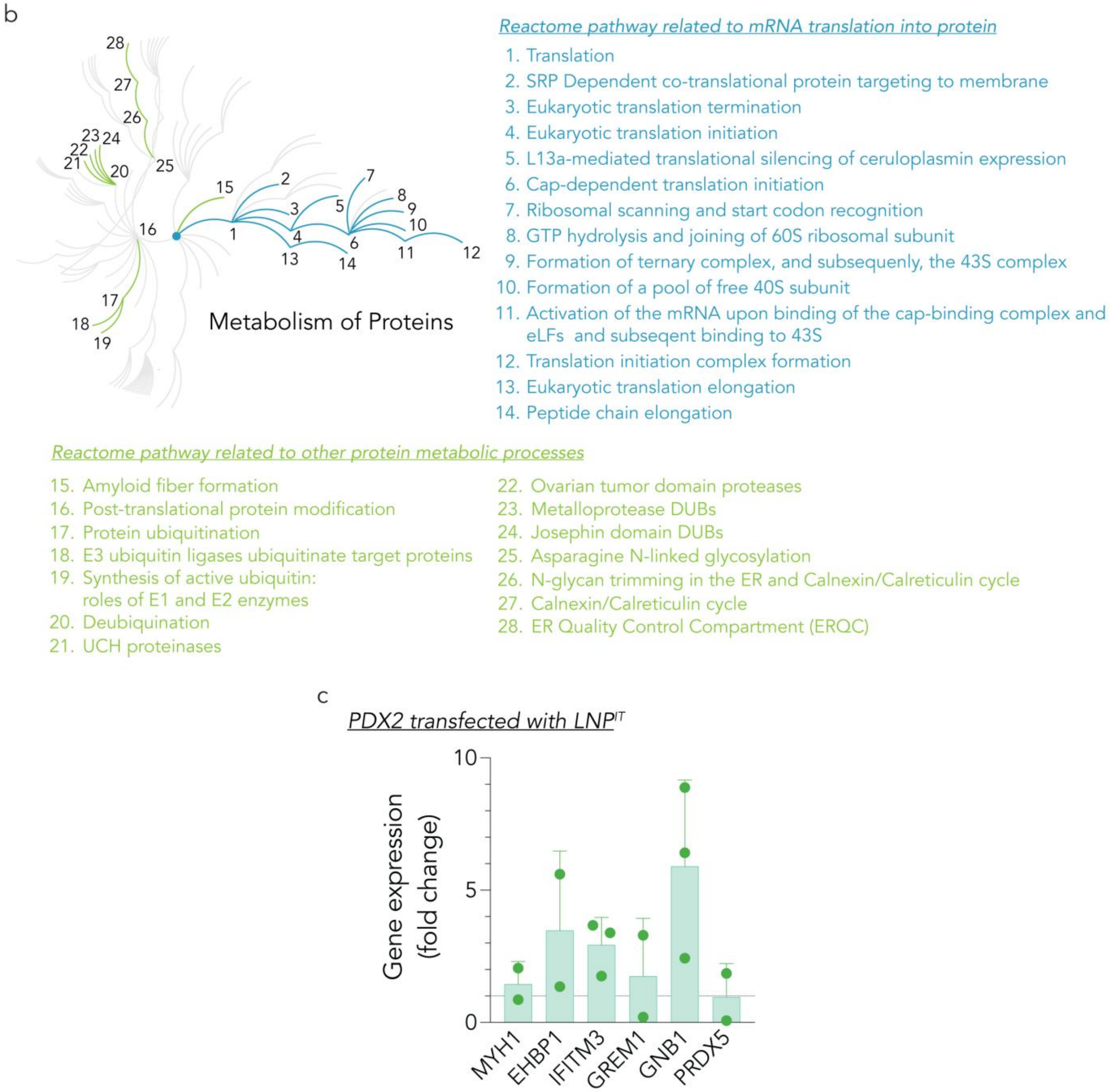

Supplementary Fig. 6 | Reactome pathway analysis for scRNA-seq data from LNP^{IT}. a, Differential gene expression of PDX tumors transfected with LNP^{IT} compared against PBS-treated PDX tumors, including the top 100 differential gene scores and full Reactome pathway analysis. **b,** Out of the 28 pathways upregulated by LNP^{IT} delivering aVHH mRNA to PDX HNSCC tumors, identified from the most upregulated genes, 14 of them are associated with mRNA translation into protein while the other 14 are related to other protein metabolic processes. **c,** Some of the top up-regulated genes identified *in vivo* in the PDX tumors transfected by LNP^{IT} were characterized *in vitro* in a second PDX line, PDX2, via qRT-PCR (n=2-3 experimental replicates).

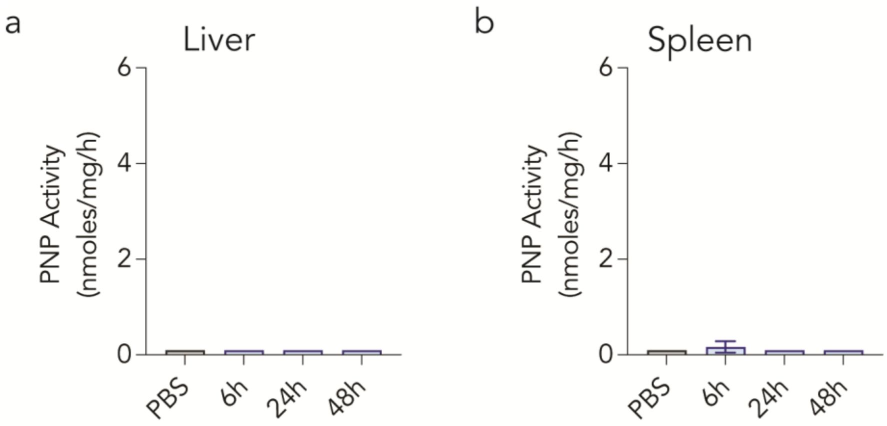

Supplementary Fig. 7 | Off-target PNP activity by LNP^{IT}. No off-target PNP activity was detected in **a,** the liver or **b,** the spleen at any timepoint following intratumoral injections of LNP^{IT} carrying PNP mRNA (n=3 experimental replicates; mean +/- SD).

a

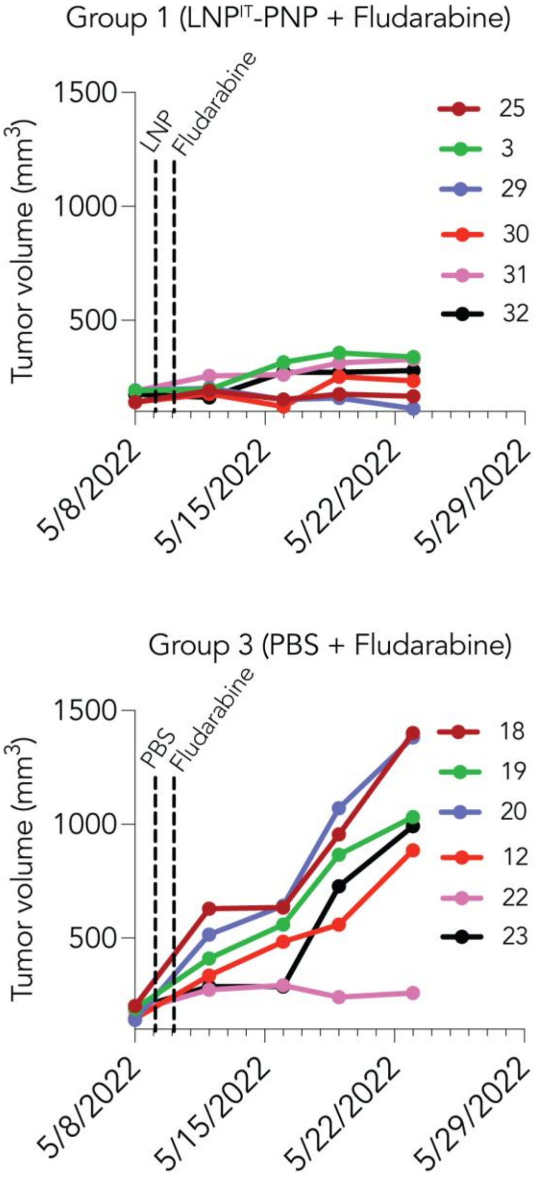
Group 1 (LNPIT-PNP + Fludarabine)
Group 2 (LNPIT-PNP + DMSO)
Group 3 (PBS + Fludarabine)
Group 4 (PBS + DMSO)
Tumor volume (mm3)
LNP
Fludarabine
DMSO
PBS
1500
1000
500
5/8/2022
5/15/2022
5/22/2022
5/29/2022
25
3
29
30
31
32
13
14
15
11
16
21
18
19
20
12
22
23
4
5
27
6
7
10

b

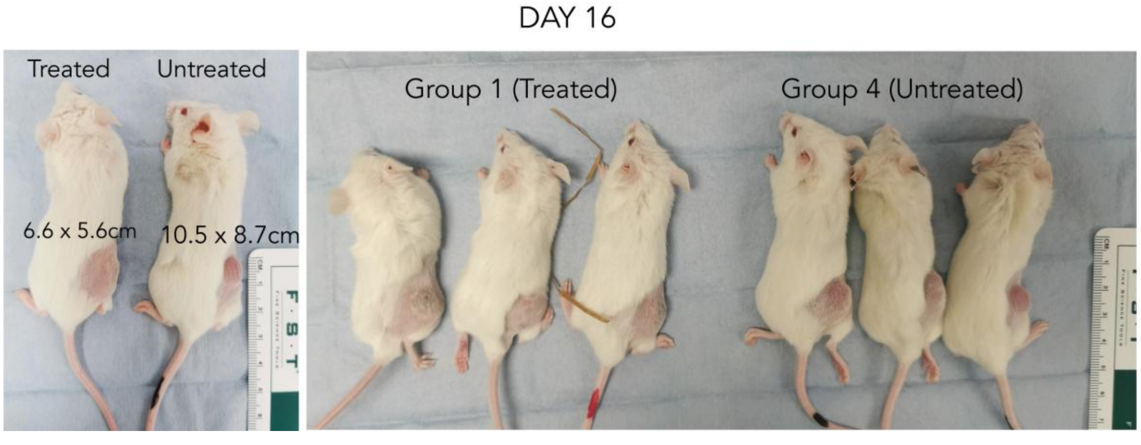
DAY 16
Treated
Untreated
6.6 x 5.6cm
10.5 x 8.7cm
Group 1 (Treated)
Group 4 (Untreated)

c

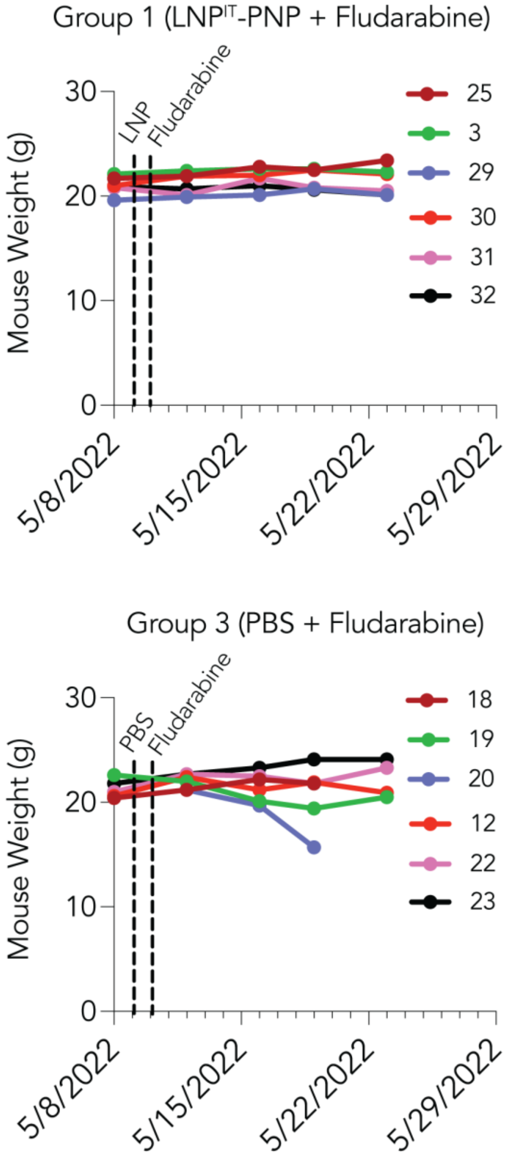

Supplementary Fig. 8 | Tumor volumes and body weights of PDX-carrying NSG mice treated with LNP^{IT} carrying PNP mRNA combined with fludarabine. PDX tumors were inoculated in NSG mice. On day 14, LNP^{IT}-PNP (or PBS for control groups) was injected at 20 µg/tumor in the morning and in the afternoon (40 µg total). The next day, fludarabine (or DMSO) was injected in the morning and afternoon. **a,** Tumor volume of each individual mouse throughout the study, measured twice a week (n=6 experimental replicates). **b,** Representative mice from groups 1 and 4 showing size reduction of the treated PDX tumor on day 16. **c,** Body weight of each individual mouse throughout the study, measured twice a week (n=6 experimental replicates).

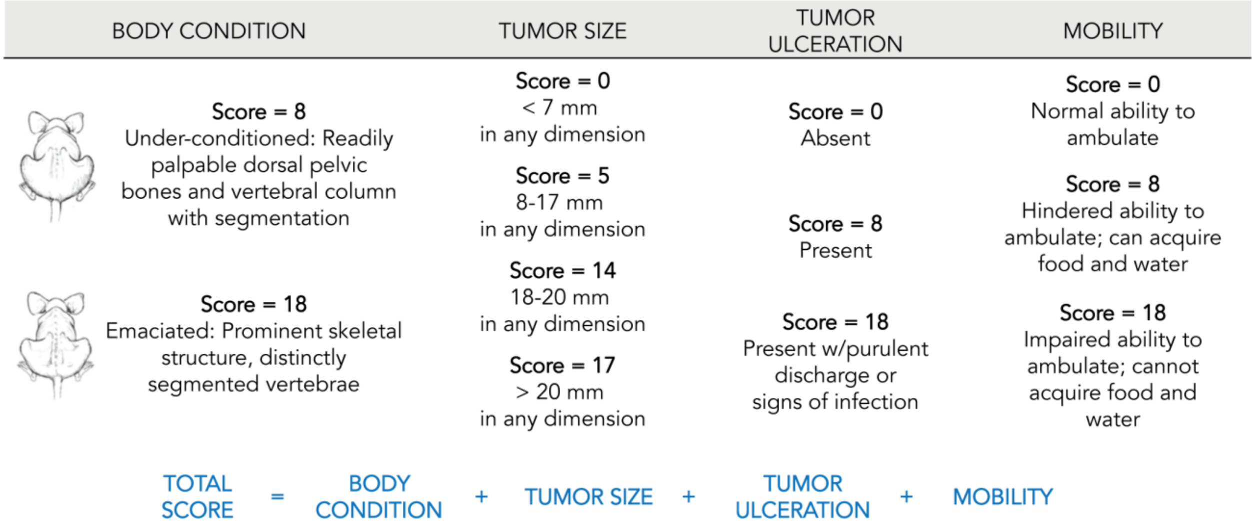

Supplementary Fig. 9 | Clinical scoring system. PDX tumors were inoculated in NSG mice. They received a first cycle of LNP^{IT}-PNP and fludarabine (or respective controls). Clinical scores were calculated based on the tumor burden (size and ulceration), body condition, and mobility. Created with BioRender.com released under a Creative Commons Attribution-NonCommercial-NoDerivs 4.0 International license (https://creativecommons.org/licenses/by-nc-nd/4.0/deed.en).

a

	PDX1	PDX2	PDX3	PDX4
Disease Body Location	Head and Neck	Head and Neck	Head and Neck	Head and Neck
OncoTree Code	HNSC	OSCS	OSCS	OSCS
Tissue Type	Resection	Resection	Resection	Resection
Tissue Collected	Neck (L)	Tongue	Tongue (Left, Lateral)	Neck (Bilateral)
Provided Tissue Origin	Primary	Primary	Primary	Primary
Gender	Male	Male	Male	Female
Human Pathogen Testing Summary	Negative	Negative	Negative	Negative
Grade/Stage Information Available	None Provided	None Provided	TMN (Pathological)	None Provided
Has Known Metastatic Disease	Not Reported	Not Reported	Yes. Tumor Grade/ Stage: pT4aN2b	Not Reported

b

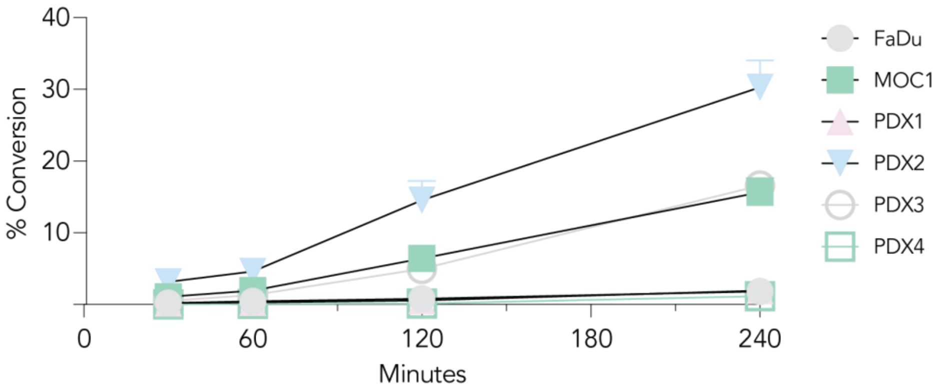

Supplementary Fig. 10 | PDX models and prodrug kinetics. a, PDX models used in this work. **b,** Conversion kinetics of fludarabine into the activated oncolytic form by LNP^{IT}-PNP in all HNSCC models studied (n=3 experimental replicates consisting of wells seeded with the respective cell line; mean +/- SD).

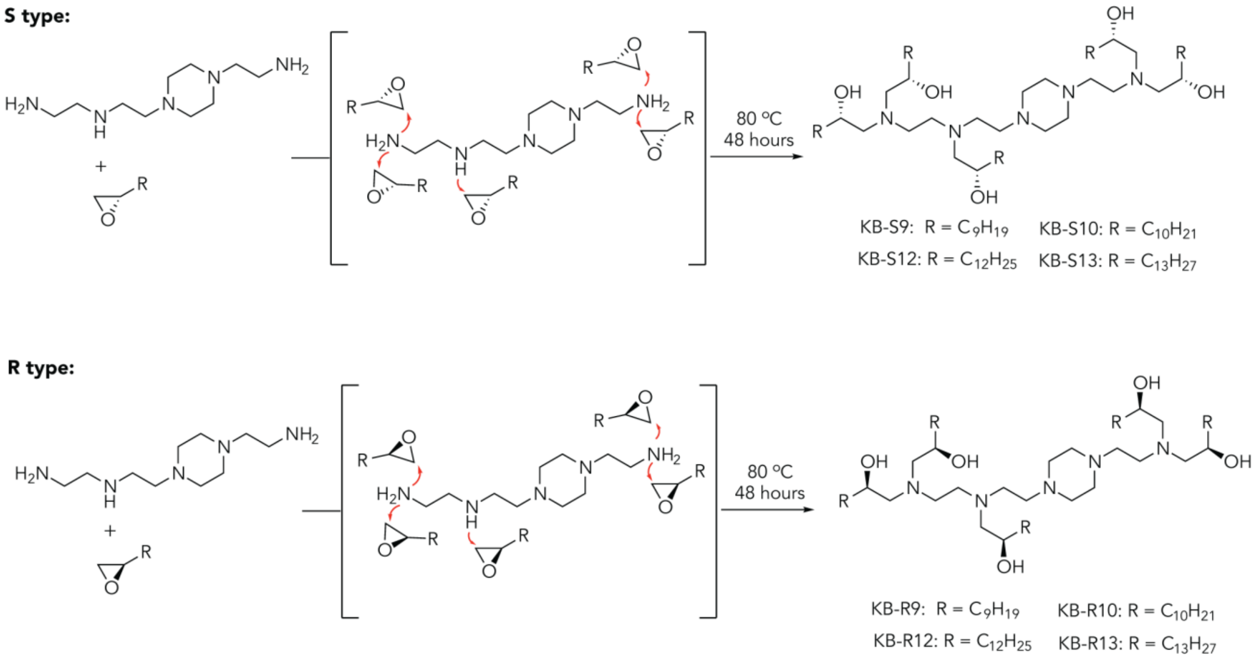

Supplementary Fig. 11 | Synthesis of C12-200 stereopure isoforms. Chemical reaction used to synthesize the stereopure isoforms of the C12-200 ionizable lipid using the respective chiral epoxides.

Supplementary Fig. 12 | NMR and HRMS data for C12-200 stereopure isoforms.

C12-200(R): ^{1}H NMR (600 MHz, $CDCl_3$) δ 3.58 (d, *J* = 38.8 Hz, 6H), 2.88 – 2.18 (m, 30H), 1.50 – 1.34 (m, 11H), 1.26 (d, *J* = 11.3 Hz, 77H), 0.88 (t, *J* = 7.0 Hz, 15H). 13C NMR (151 MHz, CDCl3) δ 70.2, 69.9, 68.4, 68.1, 67.8, 61.8, 55.7, 54.1, 35.2, 35.1, 35.1, 34.9, 32.1, 30.1, 30.01, 30.03, 29.84, 29.82, 29.80, 29.6, 29.53, 26.03, 26.0, 25.9, 22.9, 14.3. HR-ESI-MS (m/z): [M + H]+ calcd. for C70H146N5O5, 1137.1324; found, 1137.1318.

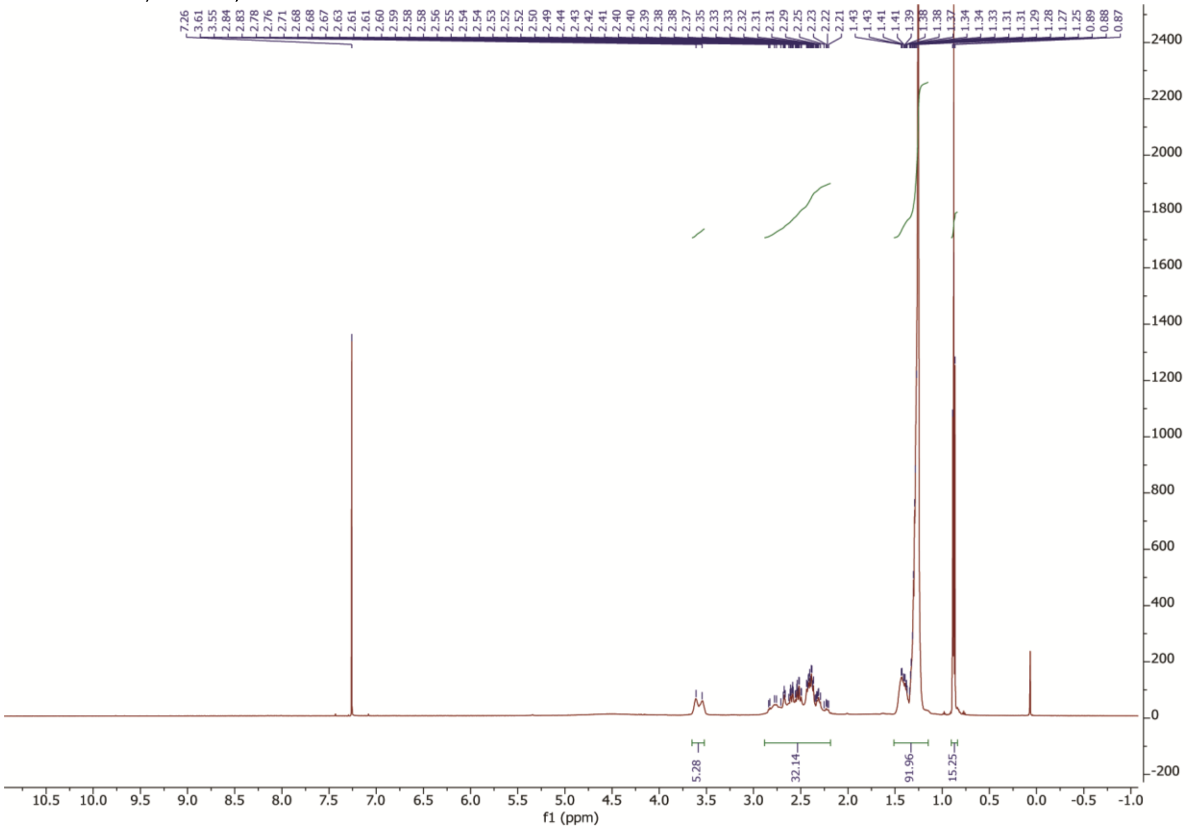

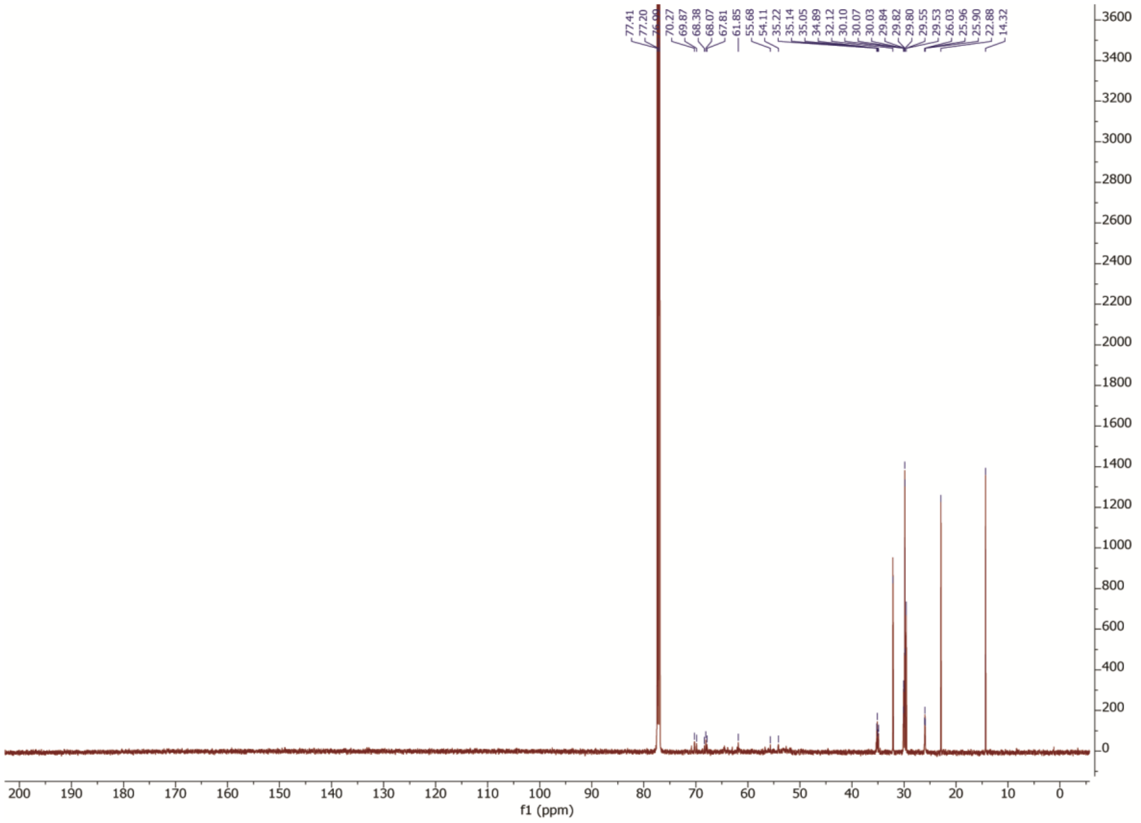

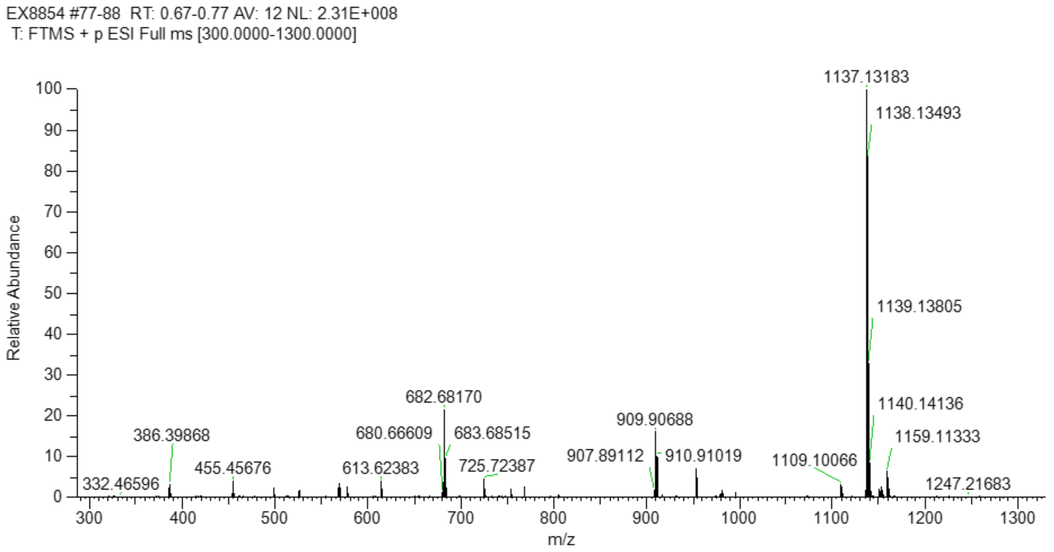

C12-200(S): 1H NMR (600 MHz, CDCl3) δ 3.59 (dd, J = 36.1, 15.8 Hz, 6H), 2.93 – 2.06 (m, 30H), 1.52 – 1.35 (m, 10H), 1.27 (d, J = 15.2 Hz, 77H), 0.88 (t, J = 7.0 Hz, 15H). 13C NMR (151 MHz, CDCl3) δ 70.3, 69.9, 68.4, 68.1, 67.8, 61.8, 55.7, 54.1, 35.3, 35.2, 35.1, 35.1, 34.9, 32.1, 30.1, 30.07, 30.03, 29.8, 29.82, 29.80, 29.55, 29.53, 26.0, 25.96, 25.90, 22.88, 14.32.HR-ESI-MS (m/z): [M + H]+ calcd. for $C_{70}H_{146}N_5O_5$, 1137.1324; found, 1137.1310.

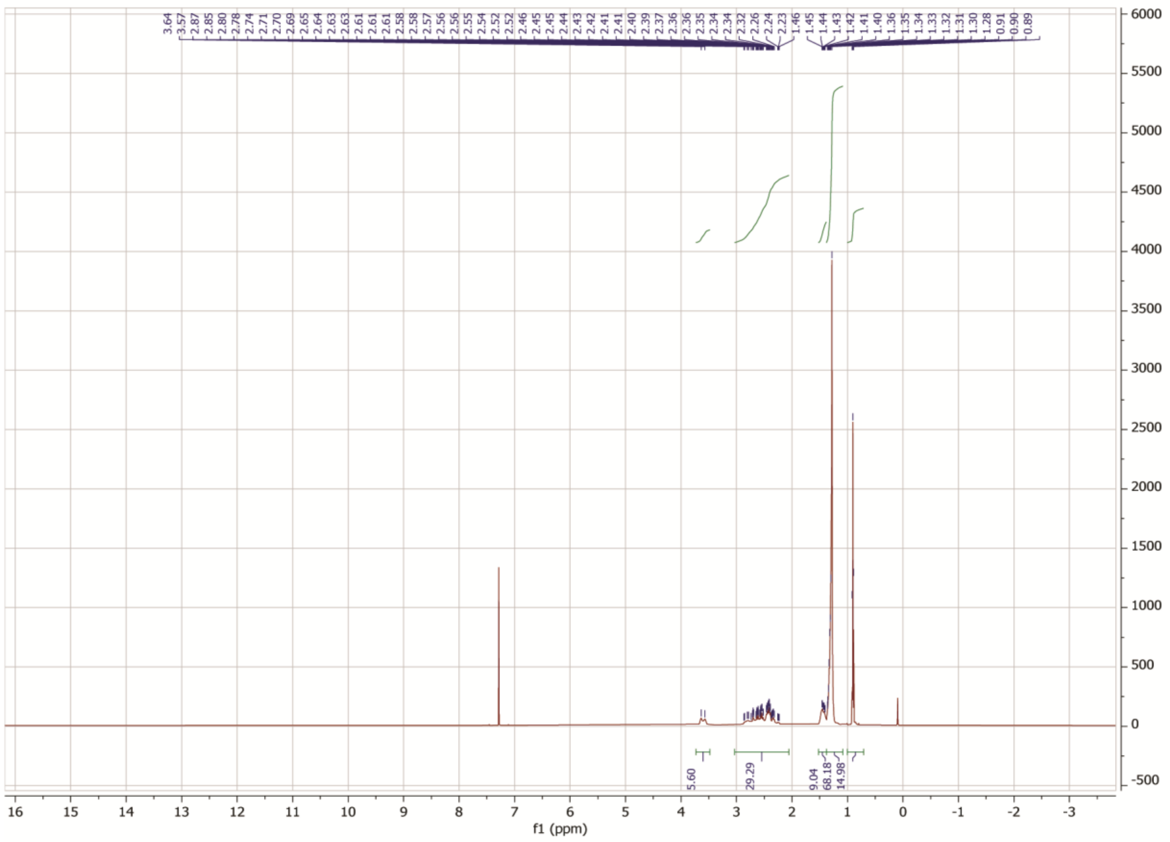

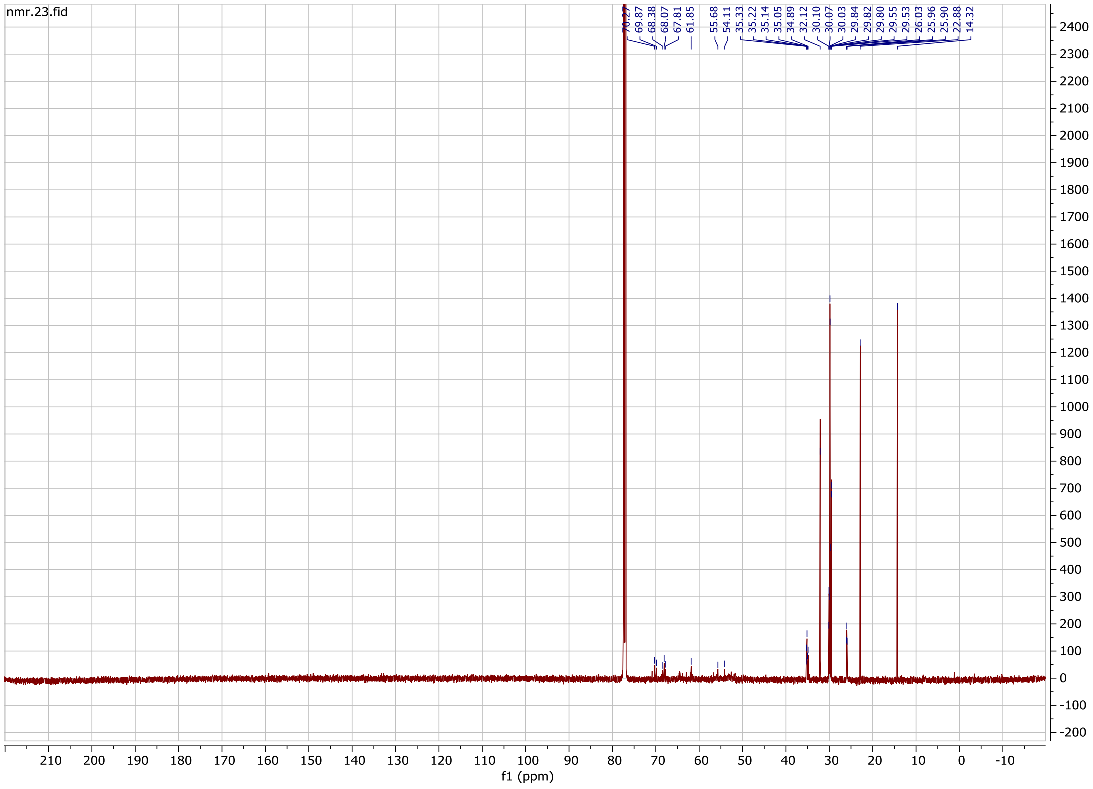

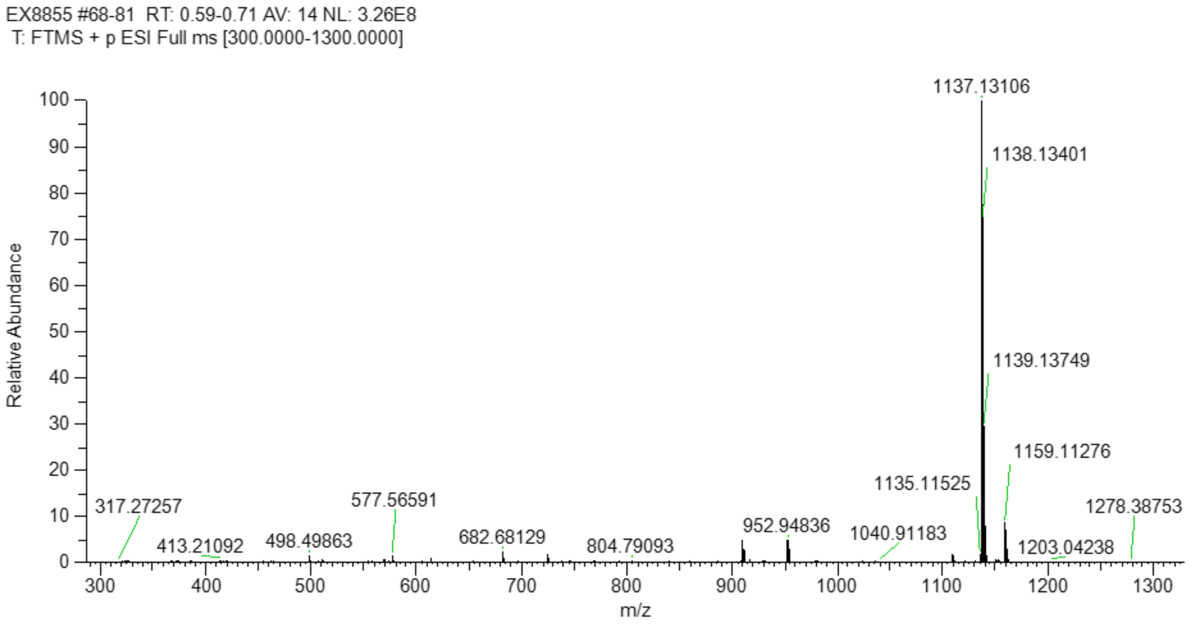
EX8855 #68-81 RT: 0.59-0.71 AV: 14 NL: 3.26E8
T: FTMS + p ESI Full ms [300.0000-1300.0000]
Relative Abundance
m/z
1137.13106
1138.13401
1139.13749
1159.11276
1135.11525
1278.38753
1203.04238
1040.91183
952.94836
804.79093
682.68129
577.56591
498.49863
413.21092
317.27257

a

Cell Type	Markers
Human HNSCC Cells	CD47+
Endothelial Cells (ECs)	CD31+
Macrophages	CD45+ CD11b+ F480+
Monocytes	CD45+ CD11b+
Neutrophils	CD45+ CD11b+ F480-
Natural Killer (NK) Cells	CD45+ NK1.1+
B Cells	CD45+ CD19+
T Cells	CD45+ CD3+

b

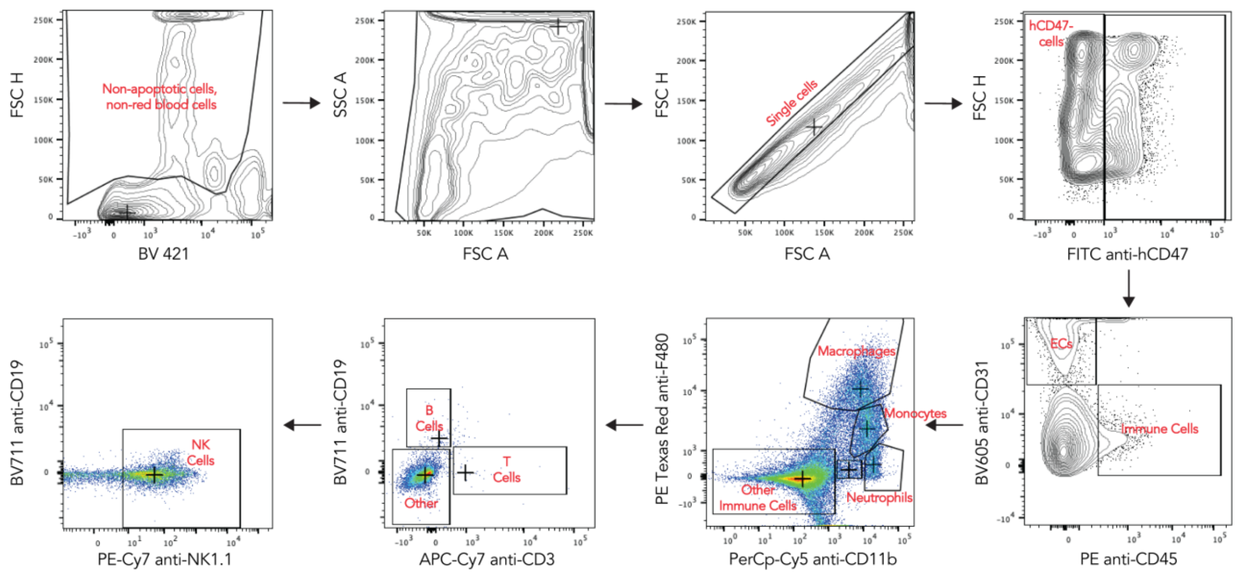

Supplementary Fig. 13 | Flow cytometry panel. a, Antibody markers used to identify the various cell types (human and murine) studied. **b,** Representative flow cytometry gates for infiltrating murine immune cells in the human HNSCC tumors (FaDu or PDX) that were identified via flow cytometry for aVHH detection.
