# Supplementary material for: Nanoparticle delivery of a prodrug-activating bacterial enzyme leads to anti-tumor responses
Source: Nat Commun. 2025 Apr 12;16:3490. doi: 10.1038/s41467-025-58548-1 (PMC11993580; doi:10.1038/s41467-025-58548-1)
Supplement: Supplementary file 2 — Reporting Summary [file 41467_2025_58548_MOESM2_ESM.pdf]

nature portfolio

Corresponding author(s): James E. Dahlman

Last updated by author(s): 3/25/2024

Reporting Summary

Nature Portfolio wishes to improve the reproducibility of the work that we publish. This form provides structure for consistency and transparency in reporting. For further information on Nature Portfolio policies, see our Editorial Policies and the Editorial Policy Checklist.

Statistics

For all statistical analyses, confirm that the following items are present in the figure legend, table legend, main text, or Methods section.

n/a	Confirmed	
☐	☒	The exact sample size (*n*) for each experimental group/condition, given as a discrete number and unit of measurement
☐	☒	A statement on whether measurements were taken from distinct samples or whether the same sample was measured repeatedly
☐	☒	The statistical test(s) used AND whether they are one- or two-sided<br>*Only common tests should be described solely by name; describe more complex techniques in the Methods section.*
☒	☐	A description of all covariates tested
☐	☒	A description of any assumptions or corrections, such as tests of normality and adjustment for multiple comparisons
☐	☒	A full description of the statistical parameters including central tendency (e.g. means) or other basic estimates (e.g. regression coefficient) AND variation (e.g. standard deviation) or associated estimates of uncertainty (e.g. confidence intervals)
☐	☒	For null hypothesis testing, the test statistic (e.g. *F*, *t*, *r*) with confidence intervals, effect sizes, degrees of freedom and *P* value noted<br>*Give P values as exact values whenever suitable.*
☒	☐	For Bayesian analysis, information on the choice of priors and Markov chain Monte Carlo settings
☒	☐	For hierarchical and complex designs, identification of the appropriate level for tests and full reporting of outcomes
☒	☐	Estimates of effect sizes (e.g. Cohen's *d*, Pearson's *r*), indicating how they were calculated

Our web collection on statistics for biologists contains articles on many of the points above.

Software and code

Policy information about availability of computer code

Data collection	BD FACSDiva software was used during flow cytometry data collection in the BD FACS Fusion.
Data analysis	FlowJo for flow cytometry data; Prism9 for data plotting; STARsolo (v 2.9.7), Seurat (v 4.0.4), DoubletFinder (v3), and BBrowserX were used for single cell RNA-sequencing data analysis (all specified in the methods section). All code used to analyze the data is available (https://github.com/Jack-Feldman/barcode_count).

For manuscripts utilizing custom algorithms or software that are central to the research but not yet described in published literature, software must be made available to editors and reviewers. We strongly encourage code deposition in a community repository (e.g. GitHub). See the Nature Portfolio guidelines for submitting code & software for further information.

Data

Policy information about availability of data

All manuscripts must include a data availability statement. This statement should provide the following information, where applicable:

- Accession codes, unique identifiers, or web links for publicly available datasets
- A description of any restrictions on data availability
- For clinical datasets or third party data, please ensure that the statement adheres to our policy

The data generated in this study are provided in the main figures or in the Supplementary Information. Source data are provided with this paper. The scRNA-seq data generated in this study have been deposited in the SRA database under BioProject ID PRJNA1090507, BioSamples accession numbers SAMN40568326 and SAMN40568327, SRA numbers SRR28411185 [https://dataview.ncbi.nlm.nih.gov/object/SRR28411185] and SRR28411184 [https://dataview.ncbi.nlm.nih.gov/

object/SRR28411184].

Research involving human participants, their data, or biological material

Policy information about studies with human participants or human data. See also policy information about sex, gender (identity/presentation), and sexual orientation and race, ethnicity and racism.

Reporting on sex and gender	For all 4 patient-derived xenograft samples used in this study, any sex and gender information available from NCI PDMR is reported in a table in Supplementary Figure 12a.
Reporting on race, ethnicity, or other socially relevant groupings	n/a
Population characteristics	For all 4 patient-derived xenograft samples used in this study, any population characteristics information available from NCI PDMR is reported in a table in Supplementary Figure 12a.
Recruitment	n/a
Ethics oversight	Patient-derived xenograft (PDX) cells were obtained as mixed/crude (non-clonal) tumor samples extracted from head and neck tumors according to protocols at the National Cancer Institute (NCI) patient-derived models repository (PDMR) (https://pdmr.cancer.gov/). All animal experiments and research performed in this study with these cells comply with all relevant ethical regulations in accordance with Emory University's IACUC.

Note that full information on the approval of the study protocol must also be provided in the manuscript.

Field-specific reporting

Please select the one below that is the best fit for your research. If you are not sure, read the appropriate sections before making your selection.

☒ Life sciences ☐ Behavioural & social sciences ☐ Ecological, evolutionary & environmental sciences

For a reference copy of the document with all sections, see nature.com/documents/nr-reporting-summary-flat.pdf

Life sciences study design

All studies must disclose on these points even when the disclosure is negative.

Sample size	At least N = 2 – 6 mice/group were used for all studies. In the case of screens and formulations, N=2-3 mice were used as control and N=3-4 mice were used for LNP-treated groups. For scRNA-seq studies, N=3 mice/group were used. In all tumor regression studies, N=6 mice were used. No sample size calculation was performed. Samples were chosen in at least triplicates for in vivo RNA delivery and protein expression studies, and N=6 sample sizes were used in all tumor regression studies for sufficient conviction on the therapeutic efficacy.
Data exclusions	No data were excluded from the analyses.
Replication	Any LNP winners from the screen were confirmed multiple times using various different mNRA payloads, different quantification assays (IVIS, Flow Cytometry, scRNA-seq, HPLC), and different tumor models. The tumor regression studies were also performed more than once to ensure the anti-tumor responses were reproducible. In specific, in vivo tumor regression studies in the patient-derived xenograft (PDX) model were performed twice. The therapeutic approach was also evaluated once in FaDu (second human xenograft) and once in MOC1 (syngeneic murine model).
Randomization	Mice were completely randomized among control and treated groups prior to any study. Once tumor sizes reached between 150 and 250 mm^3 in size, animals were divided into experimental groups and treated accordingly for all tumor regression studies.
Blinding	No blinding from the investigator side because we had 3 different control groups to evaluate each component of the prodrug combination, so we wanted to be able to identify any component-specific toxicities, if any, that emerged.

Reporting for specific materials, systems and methods

We require information from authors about some types of materials, experimental systems and methods used in many studies. Here, indicate whether each material, system or method listed is relevant to your study. If you are not sure if a list item applies to your research, read the appropriate section before selecting a response.

Materials & experimental systems

n/a	Involved in the study
	☒ Antibodies
	☒ Eukaryotic cell lines
☒	☐ Palaeontology and archaeology
	☒ Animals and other organisms
☒	☐ Clinical data
☒	☐ Dual use research of concern
☒	☐ Plants

Methods

n/a	Involved in the study
☒	☐ ChIP-seq
	☒ Flow cytometry
☒	☐ MRI-based neuroimaging

Antibodies

Antibodies used

For flow cytometry, the antibody clones used were anti-CD31 (390, BioLegend), anti-CD45.2 (104, BioLegend), anti-hCD47 (CC2C6, BioLegend), and MonoRabTM rabbit Anti-Camelid VHH Antibody iFluor647 (A01994, GenScript), all at 1:500 dilution ratios in 10% fetal bovine serum (FBS) in PBS solutions were the single-cell suspension samples were dissolved for flow cytometry.
For scRNA-seq, tagging was done using TotalSeqTM anti-human Hashtag antibody (5 μg/mL; [TotalSeq-A0251 (BioLegend 394601), TotalSeq-A0252 (BioLegend 394603), TotalSeq-A0253 (BioLegend 394605), TotalSeq-A0257 (BioLegend 394613), TotalSeq-A0258 (BioLegend 394615), TotalSeq-A0259 (BioLegend 394617)]) and oligo-tagged anti-VHH antibody (5 μg/mL; A01860, Genscript).

Validation

All antibodies satisfy the manufacturer's validation standards. Information below.

For flow cytometry:

1. FITC anti-human CD47 antibody
Catalog: 323106 (BioLegend)
Verified reactivity: Human
Reported reactivity: African Green, Baboon, Cynomolgus, Rhesus
Application: Flow cytometry – quality tested
Recommended usage: Each lot of this antibody is quality control tested by immunofluorescent staining with flow cytometric analysis. For flow cytometric staining, the suggested use of this reagent is 5 μl per million cells in 100 μl staining volume or 5 μl per 100 μl of whole blood.
Technical Data Sheet: https://d1spbj2x7qk4bg.cloudfront.net/de-at/products/fitc-anti-human-cd47-antibody-3707?pdf=true&displayInline=true&leftRightMargin=15&topBottomMargin=15&filename=FITC%20anti-human%20CD47%20Antibody.pdf&v=20240208073156

2. MonoRab™ Rabbit Anti-Camelid VHH Antibody [iFluor 647], mAb
Catalog: A01994 (GenScript)
Verified reactivity: Camelid VHH (Alpaca, Llama, and Camel)
Application: Flow cytometry
Recommended usage: 1:500 – 1:2,000
Technical Data Sheet: https://www.genscript.com/antibody/A01994-MonoRab_Rabbit_Anti_Camelid_VHH_Antibody_iFluor_647_mAb.html

For scRNA-seq:

1. TotalSeq-A anti-human Hashtag antibodies:
a) TotalSeq-A0251 (BioLegend 394601)
b) TotalSeq-A0252 (BioLegend 394603)
c) TotalSeq-A0253 (BioLegend 394605)
d) TotalSeq-A0257 (BioLgened 394613)
e) TotalSeq-A0258 (BioLgened 394615)
f) TotalSeq-A0259 (BioLgened 394617)
Clone: LNH-94; 2M2
Verified reactivity: Human
Application: Proteogenomics – Quality tested
Recommended usage: For Proteogenomics analysis, the suggested starting amount of this reagent for titration is ≤ 1.0 μg per million cells in 100 μL volume.
Technical Data Sheets:
a) https://d1spbj2x7qk4bg.cloudfront.net/fr-ch/products/totalseq-a0251-anti-human-hashtag-1-16080?pdf=true&displayInline=true&leftRightMargin=15&topBottomMargin=15&filename=TotalSeq%E2%84%A2-A0251%20anti-human%20Hashtag%201%20Antibody.pdf&v=20240208073156
b) https://d1spbj2x7qk4bg.cloudfront.net/fr-ch/products/totalseq-a0252-anti-human-hashtag-2-antibody-16081?pdf=true&displayInline=true&leftRightMargin=15&topBottomMargin=15&filename=TotalSeq%E2%84%A2-A0252%20anti-human%20Hashtag%202%20Antibody.pdf&v=20240208073156
c) https://d1spbj2x7qk4bg.cloudfront.net/fr-ch/products/totalseq-a0253-anti-human-hashtag-3-antibody-16084?

pdf=true&displayInline=true&leftRightMargin=15&topBottomMargin=15&filename=TotalSeq%E2%84%A2-A0253%20anti-human%20Hashtag%203%20Antibody.pdf&v=20240208073156
d) https://d1spbj2x7qk4bg.cloudfront.net/fr-ch/products/totalseq-a0257-anti-human-hashtag-7-antibody-16090?pdf=true&displayInline=true&leftRightMargin=15&topBottomMargin=15&filename=TotalSeq%E2%84%A2-A0257%20anti-human%20Hashtag%207%20Antibody.pdf&v=20240208073156
e) https://d1spbj2x7qk4bg.cloudfront.net/fr-ch/products/totalseq-a0258-anti-human-hashtag-8-antibody-16092?pdf=true&displayInline=true&leftRightMargin=15&topBottomMargin=15&filename=TotalSeq%E2%84%A2-A0258%20anti-human%20Hashtag%208%20Antibody.pdf&v=20240208073156
f) https://d1spbj2x7qk4bg.cloudfront.net/fr-ch/products/totalseq-a0259-anti-human-hashtag-9-antibody-16093?pdf=true&displayInline=true&leftRightMargin=15&topBottomMargin=15&filename=TotalSeq%E2%84%A2-A0259%20anti-human%20Hashtag%209%20Antibody.pdf&v=20240208073156

2. MonoRab™ Rabbit Anti-Camelid VHH Antibody, mAb
Catalog: A01860 (GenScript)
Verified reactivity: Camelid VHH (Alpaca, Llama, and Camel)
Application: Western Blot, ELISA, Sandwich ELISA
Recommended usage: 1:1000 – 1:5000 (WB), 1:5000 – 1:10000 (ELISA), A01860 (C) - A02014 (D) (Sandwich ELISA)
Technical Data Sheet: https://www.genscript.com/product/documents?cat_no=A01860&catalogtype=Document-PROTOCOL

Eukaryotic cell lines

Policy information about cell lines and Sex and Gender in Research

Cell line source(s)	FaDu cells were obtained from the Sorscher Lab at Emory University. Patient-derived PDX cells were obtained from the Sorscher Lab too, who obtained these cells from the National Cancer Institute (NCI) patient-derived models repository (PDMR) (https://pdmr.cancer.gov/).
Authentication	Autherntication was performed on FaDu and PDX cell lines as required by Emory University School of Medicine's IACUC and the National Cancer Institute (NCI), in the case of the PDX cells via morphology and PCR assays with species-specific primers.
Mycoplasma contamination	All cell lines tested negative for mycoplasma contamination, as required by Emory University School of Medicine's IACUC.
Commonly misidentified lines (See ICLAC register)	No commonly misidentified cell lines were used in this study.

Animals and other research organisms

Policy information about studies involving animals; ARRIVE guidelines recommended for reporting animal research, and Sex and Gender in Research

Laboratory animals	All animal experiments were performed in accordance with Emory University's IACUC. All animals were housed in the Emory University Winship Cancer Center Animal Facility. NU/J mice (Jax Laboratories, strain 002019, 6-8 weeks, female), NOD.Cg-Prkdcscid, IL2rgtm1Wjl/SzJ (NSG) mice (Jax Laboratories, strain 005557, 6-8 weeks, female), and C57BL6/J mice (Jax Laboratories, strain 000664, 6-8 weeks, female) were used throughout this article. At least N = 2 – 6 female mice/group were used for all studies, unless noted otherwise. NSG and NU/J mice were housed in a microisolator on static racks in animal rooms maintained at 22 ± 2 °C, and 40-50% humidity, with 12-hour light/dark cycle. Both NSG and NU/J mice were fed with sterile Rodent Lab Diet (Laboratory Rodent Diet no. 5R53) and sterile water. The C57BL/6J mice were housed in a microisolator in animal rooms maintained at 22 ± 2 °C, and 40-50% humidity, with 12-hour light/dark cycle. C57BL/6J mice were fed with Rodent Lab Diet (Laboratory Rodent Diet no. 5053).
Wild animals	No wild animals were used in this study.
Reporting on sex	Female mice were used in all experiments.
Field-collected samples	No field-collected samples were used in this study.
Ethics oversight	All animal experiments were performed in accordance with Emory University's IACUC.

Note that full information on the approval of the study protocol must also be provided in the manuscript.

Flow Cytometry

Plots

Confirm that:

- [x] The axis labels state the marker and fluorochrome used (e.g. CD4-FITC).
- [x] The axis scales are clearly visible. Include numbers along axes only for bottom left plot of group (a 'group' is an analysis of identical markers).
- [x] All plots are contour plots with outliers or pseudocolor plots.
- [x] A numerical value for number of cells or percentage (with statistics) is provided.

Methodology

Sample preparation	Cells were isolated from digested tissues, which were harvested 16 hours after intratumoral injection with LNPs, unless otherwise noted. Mice were perfused with 20 mL of 1X PBS through the right atrium when harvesting non-tumor tissues. Tumors or livers were finely minced and transferred into a digestive enzyme solution with collagenase type I (Sigma-Aldrich), collagenase IV (Sigma-Aldrich), collagenase XI (Sigma-Aldrich), and hyaluronidase (Sigma-Aldrich), and incubated at 37 ºC with controlled shaking at 550 rpm for 45 minutes. Cell suspensions were then filtered through a 70 µm mesh. Next, cells were stained to identify specific cell populations and gate out lysed cells and red blood cells. The target CD47+ aVHH+ cell populations were then sorted using the BD FACS Fusion cell sorters in the Georgia Institute of Technology Cellular Analysis Core. The antibody clones used were anti-CD31 (390, BioLegend), anti-CD45.2 (104, BioLegend), anti-hCD47 (CC2C6, BioLegend), and MonoRabTM rabbit Anti-Camelid VHH Antibody iFluor647 (A01994, GenScript).
Instrument	BD FACS Fusion cell sorter
Software	BD FACSDiva software was used during flow cytometry data collection in the BD FACS Fusion. FlowJo was used for analyzing the data collected.
Cell population abundance	Cell populations were determined, as indicated in the Methods section, after gating out life/dead positive populations and red blood cells.
Gating strategy	Representative flow gates and specific antibody panels with gating strategies can be found in Supplementary Fig. 2, Supplementary Fig. 12.

- [x] Tick this box to confirm that a figure exemplifying the gating strategy is provided in the Supplementary Information.
